# Human Atrial Arrhythmogenesis and Sinus Bradycardia in *KCNQ1*-Linked Short QT Syndrome: Insights From Computational Modelling

**DOI:** 10.3389/fphys.2018.01402

**Published:** 2018-10-04

**Authors:** Dominic G. Whittaker, Michael A. Colman, Haibo Ni, Jules C. Hancox, Henggui Zhang

**Affiliations:** ^1^School of Biomedical Sciences, Faculty of Biological Sciences, University of Leeds, Leeds, United Kingdom; ^2^Biological Physics Group, School of Physics and Astronomy, The University of Manchester, Manchester, United Kingdom; ^3^Department of Pharmacology, University of California, Davis, Davis, CA, United States; ^4^School of Physiology, Pharmacology and Neuroscience, and Cardiovascular Research Laboratories, School of Medical Sciences, University of Bristol, Bristol, United Kingdom; ^5^School of Computer Science and Technology, Harbin Institute of Technology, Harbin, China; ^6^Space Institute of Southern China, Shenzhen, China; ^7^Key Laboratory of Medical Electrophysiology, Ministry of Education, Collaborative Innovation Center for Prevention and Treatment of Cardiovascular Disease/Institute of Cardiovascular Research, Southwest Medical University, Luzhou, China

**Keywords:** anti-arrhythmic, arrhythmia, short QT syndrome, atrial fibrillation, KCNQ1 mutations, sinus bradycardia, quinidine

## Abstract

Atrial fibrillation (AF) and sinus bradycardia have been reported in patients with short QT syndrome variant 2 (SQT2), which is underlain by gain-of-function mutations in *KCNQ1* encoding the α subunit of channels carrying slow delayed rectifier potassium current, *I*_Ks_. However, the mechanism(s) underlying the increased atrial arrhythmogenesis and impaired cardiac pacemaking activity arising from increased *I*_Ks_ remain unclear. Possible pharmacological interventions of AF in the SQT2 condition also remain to be elucidated. Using computational modelling, we assessed the functional impact of SQT2 mutations on human sinoatrial node (SAN) pacemaking, atrial repolarisation and arrhythmogenesis, and efficacy of the anti-arrhythmic drug quinidine. Markov chain formulations of *I*_Ks_ describing two *KCNQ1* mutations – V141M and V307L – were developed from voltage-clamp experimental data and then incorporated into contemporary action potential (AP) models of human atrial and SAN cells, the former of which were integrated into idealised and anatomically detailed tissue models. Both mutations shortened atrial AP duration (APD) through distinct *I*_Ks_ ‘gain-of-function’ mechanisms, whereas SAN pacemaking rate was slowed markedly only by the V141M mutation. Differences in APD restitution steepness influenced re-entry dynamics in tissue – the V141M mutation promoted stationary and stable spiral waves whereas the V307L mutation promoted non-stationary and unstable re-entrant waves. Both mutations shortened tissue excitation wavelength through reduced effective refractory period but not conduction velocity, which served to increase the lifespan of re-entrant excitation in a 3D anatomical human atria model, as well as the dominant frequency (DF), which was higher for the V141M mutation. Quinidine was effective at terminating arrhythmic excitation waves associated with the V307L but not V141M mutation, and reduced the DF in a dose-dependent manner under both mutation conditions. This study provides mechanistic insights into different AF/bradycardia phenotypes in SQT2 and the efficacy of quinidine pharmacotherapy.

## Introduction

The short QT syndrome (SQTS) is a rare but important cardiac disorder characterised by a shortened QT interval, increased incidence of ventricular and atrial arrhythmias, and risk of sudden death in affected patients (Schimpf et al., 2005). Variants 1–3 of the SQTS are caused by gain-of-function mutations to genes encoding different K^+^ channel subunits, which carry currents responsible for phase 3 repolarisation of the cardiac action potential (AP) (Hancox et al., 2018). Mutations to the *KCNQ1* (*KvLQT1*, *Kv7.1*) gene product which, along with auxiliary subunits encoded by *KCNE1*, encodes slow delayed rectifier potassium current, *I*_Ks_, underlie SQTS variant 2 (SQT2). To date, four missense mutations have been identified in *KCNQ1*-linked SQTS: V307L (Bellocq et al., 2004), V141M (Hong et al., 2005), R259H (Wu et al., 2015), and F279I (Moreno et al., 2015), all of which result in a gain-of-function of *I*_Ks_.

There is mounting evidence for a role of *I*_Ks_ in atrial arrhythmogenesis (Christophersen and Ellinor, 2016). Upregulation of *I*_Ks_ has been identified in patients with chronic atrial fibrillation (AF) (Caballero et al., 2010; González de la Fuente et al., 2013), and mutations to *KCNQ1* have been shown to underlie lone AF (Chen et al., 2003; Lundby et al., 2007; Das et al., 2009). These findings suggest that enhanced *I*_Ks_ may play a role in the pathogenesis of AF. In some SQT2 patients, cardiac arrhythmia including AF has been reported (Hong et al., 2005; Villafañe et al., 2014; Righi et al., 2016). However, the mechanism(s) underlying atrial arrhythmogenesis and maintenance arising from increased potassium channel currents, including *I*_Ks_, remain to be fully established. The SQTS thus represents a valuable paradigm for investigating the role of K^+^ channels in AF.

Atrial fibrillation can be the first clinical presentation of the SQTS, particularly in patients diagnosed with lone AF (Hasdemir, 2016). Furthermore, the incidence of AF has been reported to be higher in patients with SQT2 than other forms of SQTS (63% vs. 21%, *p* = 0.012 (Harrell et al., 2015)). The first reported SQT2 mutation, V307L KCNQ1 (Bellocq et al., 2004), was shown to shift the voltage-dependence of *KCNQ1 + KCNE1* activation toward less depolarised potentials and accelerate channel activation, causing a gain-of-function to *I*_Ks_. The proband presented with a shortened QTc interval and idiopathic ventricular fibrillation (Bellocq et al., 2004) – whether or not this mutation is able also to promote AF is not yet known. The subsequently discovered V141M KCNQ1 mutation differs from the V307L KCNQ1 mutation in that it induces an instantaneous, voltage-independent K^+^-selective current component (Hong et al., 2005). This form of SQT2 is associated with abnormally short QT intervals in affected patients, as well as multiple reports of a mixed AF and sinus bradycardia phenotype (Hong et al., 2005; Villafañe et al., 2014; Righi et al., 2016).

At present, there are no phenotypically accurate experimental models of genetic forms of SQTS. Computational modelling offers a viable way of investigating how SQTS-linked K^+^ channelopathies affect organ scale electrical propagation and arrhythmogenesis. Previous studies have investigated the functional impact of SQT2 variants on ventricular arrhythmogenesis (Zhang et al., 2008; Adeniran et al., 2017), which can be attributed to abbreviated ventricular AP duration (and therefore excitation wavelength), increased transmural dispersion of repolarisation, and increased vulnerability of tissue to initiation of unidirectional conduction block. However, the mechanism(s) by which SQT2 variants promote atrial arrhythmogenesis has not been elucidated – it is possible that the interaction between genetic mutations and intrinsic electrical heterogeneities in the human atria may form an important determinant of arrhythmogenic mechanisms, as shown in previous studies (Colman et al., 2017; Whittaker et al., 2017b). Consequently, the first aim of this study was to dissect underlying mechanisms of increased susceptibility to development of AF associated with SQT2 using both idealised and anatomically-detailed, heterogeneous multi-scale tissue models of human atrial electrophysiology. We chose to study the V141M KCNQ1 mutation, which has been linked with multiple reports of AF (Hong et al., 2005; Villafañe et al., 2014; Righi et al., 2016), and use the V307L KCNQ1 mutation as a comparator, which has distinct kinetics and has been demonstrated to promote ventricular arrhythmogenesis in our previous computational modelling study (Adeniran et al., 2017).

Effective pharmacological management of AF in KCNQ1-linked SQTS conditions is an unmet challenge. Quinidine – an established inhibitor of SQTS mutant hERG channels (Gaita et al., 2004; Hu et al., 2017) has shown some efficacy in the ventricles in non-hERG-linked SQTS (e.g., Giustetto et al., 2011). However, its anti-AF effects in the context of SQT2 remain unclear. The second aim of this study was thus to assess the effects of quinidine on arrhythmic atrial excitation waves in SQT2 using cellular and tissue level simulations that incorporated drug binding kinetics and multi-channel pharmacology.

## Materials and Methods

### Markov Chain Models of *I*_Ks_

A Markov chain (MC) formulation of human cardiac *I*_Ks_ (Silva and Rudy, 2005; Adeniran et al., 2017) was used to simulate *I*_Ks_ in wild type (WT) and SQT2 mutation conditions (MC scheme shown in **Supplementary Figure S1**). The V307L KCNQ1 mutation MC formulation has been described and validated previously (Adeniran et al., 2017); nonetheless, for completeness, the response of WT and V307L mutant currents to simulated voltage clamps – namely the *I*–*V* relation and steady state activation – and human atrial AP clamp is shown in **Supplementary Figure S2**.

**FIGURE 1:**
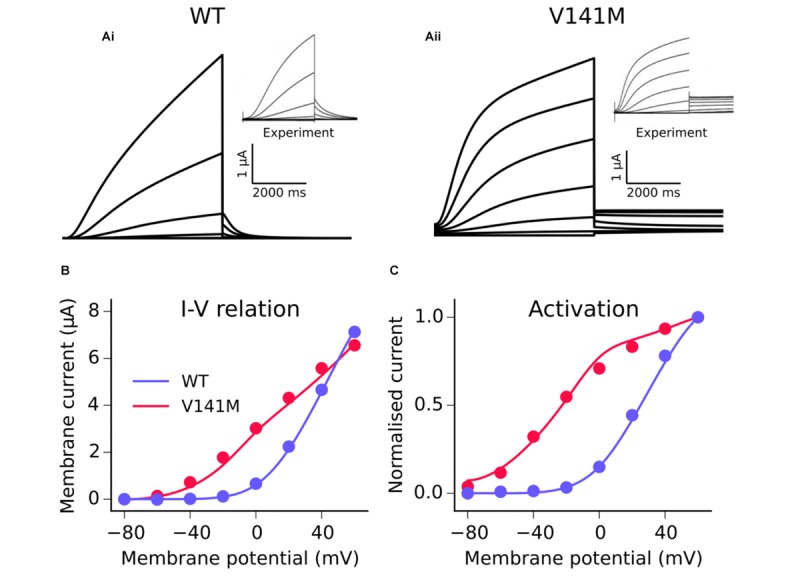
Kinetics of the V141M KCNQ1 mutant *I*_Ks_ model. A comparison of simulated voltage clamp current traces in WT **(Ai)** and V141M **(Aii)** conditions at 22°C. Experimental traces inset are reproduced with permission from Restier et al. (2008), which describes the protocols used. Simulated (solid lines) *I*–*V* relation **(B)** and voltage dependence of activation **(C)** under WT and V141M mutation conditions, compared with experimental data (points).

The MC formulation of *I*_Ks_ was subsequently employed to develop a model of the V141M mutation in *KCNQ1*, first described by Hong et al. (2005). Multi-objective fitting to experimental data (Restier et al., 2008), namely the *I*–*V* relation, steady state activation, and voltage clamp current traces, was performed using a bounded Nelder–Mead simplex algorithm (Moreno et al., 2016). As experimental data were acquired at room temperature, a Q_10_ correction value of 3.5 (Seebohm et al., 2001) was applied in order to represent kinetics at physiological temperature. An additional voltage-independent parameter, ξ, was introduced to account for the constitutively active component of *I*_Ks_ observed in *KCNQ1* V141M mutant channels (Hong et al., 2005; Restier et al., 2008). The response of V141M mutant currents to simulated voltage clamps and corresponding *I–V* relation and steady state activation is shown in **Figure 1**. The V141M mutant *I*_Ks_ formulation reproduced accurately the experimentally measured *I*–*V* relationship and voltage dependence of activation under voltage clamp conditions, as well as faster activation and slower deactivation (Restier et al., 2008). For both mutations, in order to mimic the heterozygous state of probands, a heterozygous mutation formulation consisting of 50% WT and 50% mutant subunit channels was constructed (Adeniran et al., 2017). For more details of the MC scheme, see **Supplementary Method 1.1**.

### Modelling Electrophysiology of the Human Atria and Sinoatrial Node

The Colman et al. (2013) family human atrial cell models incorporating regional heterogeneity, recently updated by Ni et al. (2017), was used to simulate human atrial electrophysiology in this study, and is hereinafter referred to as the CNZ (Colman-Ni-Zhang) model. For all single cell, 1D, and 2D simulations the baseline right atrium (RA) model was used. The equations for *I*_Ks_ – which are native to the parent Courtemanche-Ramirez-Nattel (CRN) model (Courtemanche et al., 1998) – were replaced with that of the WT MC formulation, with a current density within the range measured in human atrial myocytes (Caballero et al., 2010) – **Supplementary Figure S4**. Furthermore, as a theoretical consideration, the effects of AF-induced electrical remodelling (**Supplementary Figure S6**) were incorporated into supplementary CNZ model simulations, to assess the combined influence of *KCNQ1* mutations and AF remodelling. A recently developed human sinoatrial node (SAN) model (Fabbri et al., 2017), hereinafter referred to as the FS (Fabbri-Severi) model, was used to simulate the AP of primary pacemaker cells in the human heart. The native equations for *I*_Ks_ were replaced by the WT MC *I*_Ks_ formulation – **Supplementary Figure S7** shows the agreement between the modified FS model AP and published experimental recordings from human SAN myocytes (Verkerk et al., 2007). Full details of the CNZ and FS models, including definitions of quantitative AP biomarkers used, the effects of the new *I*_Ks_ formulation on the AP, regional human atrial electrophysiology models, AF remodelling, and parasympathetic modulation with acetylcholine (ACh), can be found in **Supplementary Method 1.2**–**1.4**.

### Modelling Pharmacological Actions of Quinidine

In our recent prior study (Whittaker et al., 2017a), state-dependent binding of quinidine to hERG and sodium channels was simulated in human ventricle models. Secondary blocking actions of quinidine on other affected ionic currents were described using a simple pore block approach. The actions of quinidine on human atrial cells in this study were represented using the same formulations and IC_50_ (half maximal inhibitory concentration) values (Whittaker et al., 2017a). Additionally, quinidine block of the atrial-specific ultra-rapid delayed rectifier potassium current, *I*_Kur_, was incorporated into CNZ simulations with an IC_50_ of 6.6 μM (Nenov et al., 1998), as measured in human atrial myocytes. Concentrations of 1, 2, and 5 μM quinidine, which likely represent realistic maximal unbound concentrations (Whittaker et al., 2017a), were used. A summary of IC_50_ values for quinidine inhibition of multiple ion channels is given in **Supplementary Table S4**. Further details regarding kinetics, parameters, and equations can be found in **Supplementary Method 1.5**, as well as our previous study (Whittaker et al., 2017a).

### Tissue Simulations

The effects of SQT2 mutations on human atrial electrophysiology were further investigated using a hierarchy of tissue models. One-dimensional (1D) models of human atrial strands were used to assess the effects of *KCNQ1* mutations on the effective refractory period (ERP), conduction velocity (CV), and excitation wavelength (WL). In order to characterise re-entrant excitation wave dynamics, an isotropic 2D sheet of human atrial tissue was used, wherein spiral waves were initiated using an S1–S2 cross-field protocol (Whittaker et al., 2017b). In order to characterise the lifespan and dominant frequency (DF) of arrhythmic excitation waves, as well as the response to quinidine, a 3D anatomical model of the human atria (Seemann et al., 2006; Colman et al., 2013) with heterogeneity of electrophysiology, rule-based fibre orientations (Krueger et al., 2011), and validated activation times was used (**Supplementary Figure S10**). Scroll waves were initiated proximal to the SAN in the RA using the phase distribution method (Biktashev and Holden, 1998; Colman et al., 2017; Whittaker et al., 2017b), which developed into functional and/or anatomical re-entries in the 3D anatomical human atria model. The rate of electrical activation during re-entrant excitation was determined from pseudo-ECG (pECG) signals. It should be noted that the SAN region was modelled electrically as CT tissue in 3D simulations for simplicity (Colman et al., 2013, 2017). Further descriptions of tissue models and simulation protocols are given in **Supplementary Methods 1.6**–**1.9**.

## Results

### Modification of Human SAN Cell Pacemaking by SQT2 Mutant *I*_Ks_

**FIGURE 2:**
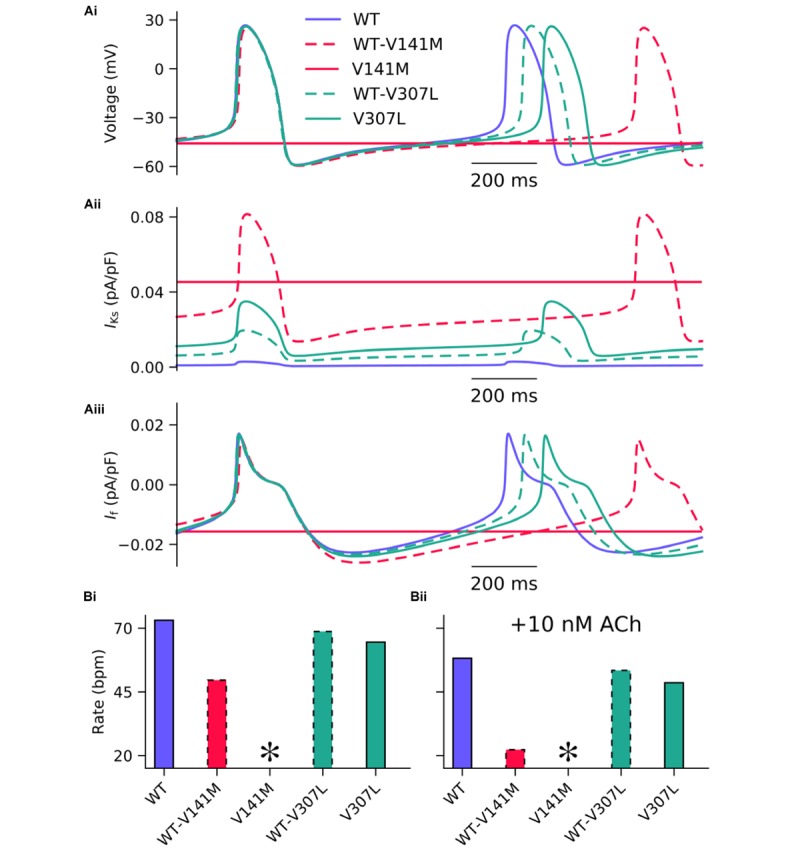
Simulated AP profiles and current traces under WT and SQT2 mutant conditions in human SAN myocytes. Spontaneous pacemaker APs under WT (lilac, solid line), WT-V141M (red, dashed line), V141M (red, solid line), WT-V307L (turquoise, dashed line), and V307L (turquoise, solid line) conditions **(Ai)** in the absence of autonomic modulation, with corresponding current traces for *I*_Ks_ **(Aii)** and *I*_f_ **(Aiii)**. The beating rate is summarised as a bar chart, without autonomic modulation **(Bi)**, and with addition of 10 nM acetylcholine (ACh) **(Bii)**. ^∗^Denotes pacemaking abolished.

The effects of *KCNQ1* V141M and V307L gain-of-function mutations on *I*_Ks_ were first investigated in the FS SAN model (**Figure 2**). The heterozygous form of the V141M mutation decreased the diastolic depolarisation rate (DDR) due to increased repolarising current at potentials negative to the take-off (threshold) potential, which extended the pacemaker potential and reduced the beating rate of single SAN cells from 73 bpm in the WT condition to 50 bpm. The homozygous condition abolished pacemaking, as increased repolarising *I*_Ks_ prevented the membrane potential from reaching the take-off potential. The APD_90_ in the WT-V141M condition was reduced from 151.4 ms in the WT condition to 143.4 ms. The effects of the V307L mutant were comparatively modest; the WT-V307L and V307L mutation conditions reduced the beating rate to 69 bpm and 64 bpm, respectively. Furthermore, the reduction in APD_90_ was less substantial, being reduced to 149.7 ms and 148.1 ms in the WT-V307L and V307L conditions, respectively.

In addition to reduced beating rate due to decreased DDR, the maximum upstroke velocity was reduced by the WT-V141M mutation condition and to a lesser extent by the homozygous form of the V307L mutation. Under simulated parasympathetic modulation by 10 nM ACh, the reduction in beating rate in V307L mutation conditions was still relatively minor (**Figure 2Bii**), whereas pacemaking rate was reduced to 22 bpm in WT-V141M mutation conditions and was abolished completely by the homozygous V141M expression condition.

### Modification of Human Atrial Action Potentials by SQT2 Mutant *I*_Ks_

**FIGURE 3:**
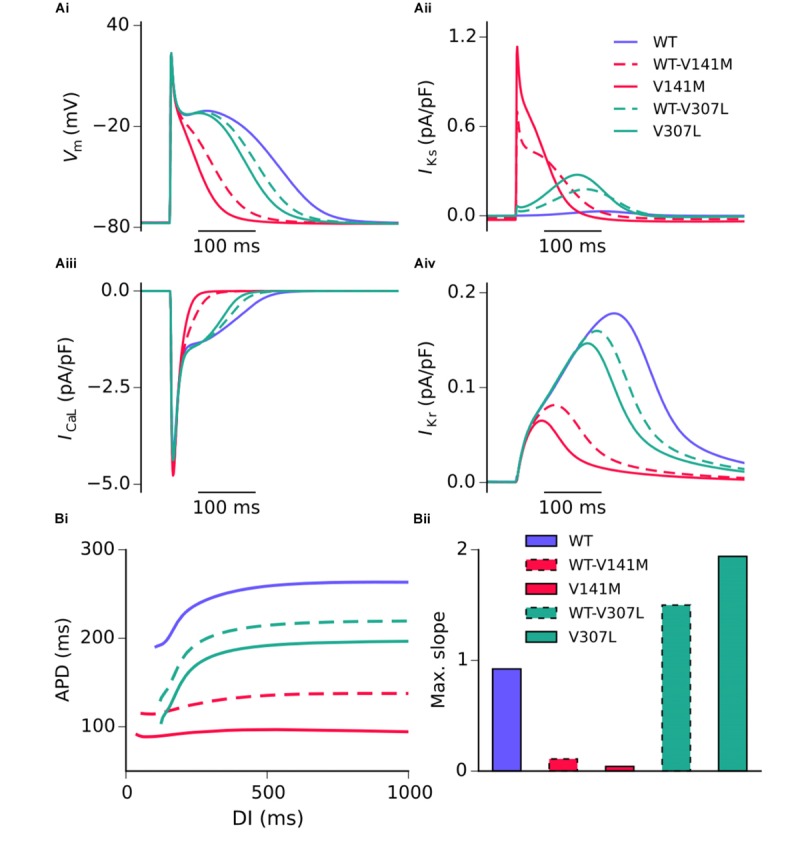
Simulated AP profiles and current traces under WT and SQT2 mutant conditions in human atrial myocytes. AP profiles in WT, WT-V141M, V141M, WT-V307L, and V307L conditions at 1 Hz **(Ai)**, as well as corresponding *I*_Ks_ **(Aii)**, *I*_CaL_ **(Aiii)**, and *I*_Kr_ **(Aiv)**. APD at –70 mV plotted against diastolic interval (DI), measured using an S1–S2 protocol **(Bi)**, and a bar chart showing maximal slope of restitution **(Bii)**.

Action potential shortening occurred under all SQT2 mutant conditions in the baseline CNZ RA model at 1 Hz (**Figure 3**), as well as other atrial sub-regions (**Supplementary Figure S5**). The V141M mutation abolished the AP plateau phase and reduced the APD_90_ from 250.0 ms in the WT condition to 124.7 ms and 85.5 ms under heterozygous and homozygous forms, respectively (**Figure 3Ai**). In contrast, the ‘spike and dome’ morphology of the AP was preserved under V307L mutant conditions, as these mutations exerted their effects mainly during the terminal phase of repolarisation. The heterozygous and homozygous forms of these mutants abbreviated the APD_90_ to 207.6 ms and 185.4 ms, respectively. The V141M mutation induces a constitutively active voltage-independent component of *I*_Ks_ (Hong et al., 2005; Restier et al., 2008). The combination of this and significantly slowed deactivation resulted in a large outward current during membrane depolarisation (**Figure 3Aii**). The V307L mutation, in contrast, augmented *I*_Ks_, but did not fundamentally change its profile.

**Figure 3** shows secondary effects of SQT2 *KCNQ1* mutants on L type calcium current, *I*_CaL_ (**Figure 3Aiii**) and rapid delayed rectifier potassium current, *I*_Kr_ (**Figure 3Aiv**) during APs. The balance of ionic currents which maintains the plateau phase was largely abolished in V141M mutation conditions, as the increase in repolarising *I*_Ks_ overpowered inward *I*_CaL_. This also had the effect of activating *I*_Kr_ to a lesser extent, thus considerably decreasing its peak current density and contribution to AP repolarisation. The effects of the V307L mutation on *I*_Ks_ were less pervasive, with the current profiles of *I*_CaL_ and *I*_Kr_ being altered to a smaller extent. Aside from the APD, alterations to other AP parameters induced by the SQT2 mutations were minimal. A summary of single cell simulations (SAN and RA) is given in **Table 1**.

**Table 1:** Single cell AP properties in WT and SQT2 mutant conditions in the FS human SAN model (autorhythmic) and CNZ human atrial cell model (paced at 1 Hz).

Sinoatrial node (SAN) cells					

	WT	WT-V141M	V141M	WT-V307L	V307L
**Beating rate (bpm)**	73.1	49.6	–	68.7	64.5
**APD_90_ (ms)**	151.4	143.4	–	149.7	148.1
**APD_50_ (ms)**	125.5	118.1	–	123.9	122.5
**MUV (V/s)**	7.5	7.2	–	7.5	7.4
**DDR (mV/s)**	35.5	21.5	–	32.6	29.9

**Atrial cells**					

	**WT**	**WT-V141M**	**V141M**	**WT-V307L**	**V307L**

**APD_90_ (ms)**	250.0	124.7	85.5	207.6	185.4
**APD_50_ (ms)**	145.9	45.7	30.1	120.0	105.4
**APA (mV)**	103.0	103.1	102.9	103.3	103.4
**MUV (V/s)**	212.4	214.7	214.6	214.2	214.4


*Abbreviations are as follows: beats per minute (bpm); action potential duration at 50% and 90% repolarisation (APD_*50*_ and APD_*90*_, respectively); maximum upstroke velocity (MUV); diastolic depolarisation rate (DDR); and action potential amplitude (APA).*

All SQT2 mutations investigated shortened the APD across a range of diastolic intervals (DIs; **Figure 3Bi**). However, the V141M and V307L KCNQ1 mutations produced opposing effects on the maximum slope of restitution. Both heterozygous and homozygous V141M mutant conditions showed almost no rate adaptation, with significantly reduced maximal slope of restitution. In contrast, the V307L mutation conditions increased the maximal slope of restitution, markedly so in the homozygous V307L condition. Furthermore, whereas the APD measured in the WT-V141M condition was considerably shorter than that measured for the V307L mutation at 1 Hz (124.7 ms vs. 185.4 ms), at fast pacing rates the restitution curves crossed over, indicating greater rate adaptation for the V307L mutation.

### Tissue Restitution Properties

**FIGURE 4:**
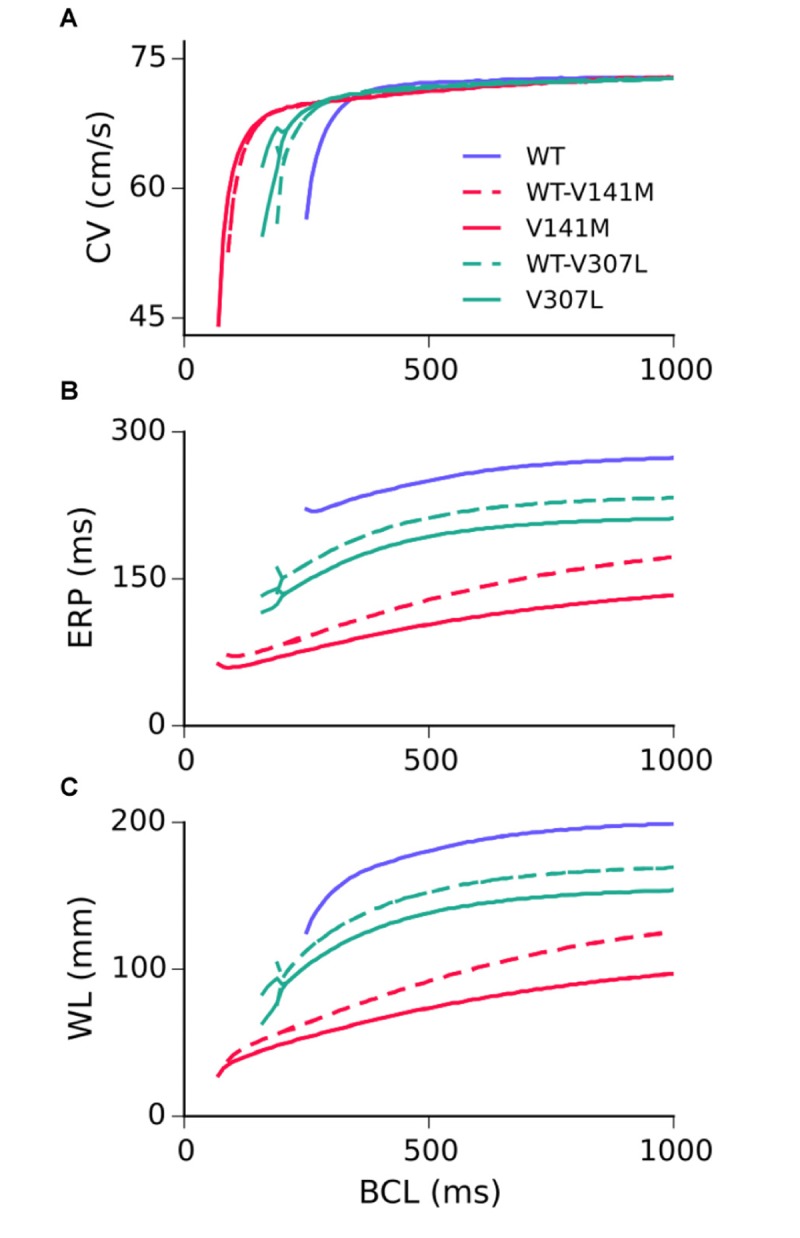
Tissue restitution properties in WT and mutation conditions. Steady-state restitution of the conduction velocity (CV) **(A)**, effective refractory period (ERP) **(B)**, and excitation wavelength (WL) **(C)** against a range of basic cycle lengths (BCL), measured in the 1D tissue model.

Restitution curves for the CV, ERP, and WL measured in the 1D strand are shown in **Figure 4**. None of the SQT2 mutation conditions investigated considerably affected the CV at pacing rates slower than 2 Hz, whereas all mutation conditions reduced the ERP across all basic cycle lengths (BCLs) investigated. As the excitation WL is given by CV × ERP, the WL was thus also decreased by SQT2 mutations across all BCLs, indicating that higher frequency excitations can be supported by SQT2 mutant tissue (investigated in **Scroll wave dynamics in 3D anatomical human atria geometry**). The V307L mutation conditions (the homozygous form in particular) induced beat-to-beat AP alternans at fast pacing rates, which was evident in restitution curves for the CV, ERP, and WL.

### Spiral Wave Dynamics in Idealised 2D Sheets of Human Atrial Tissue

**FIGURE 5:**
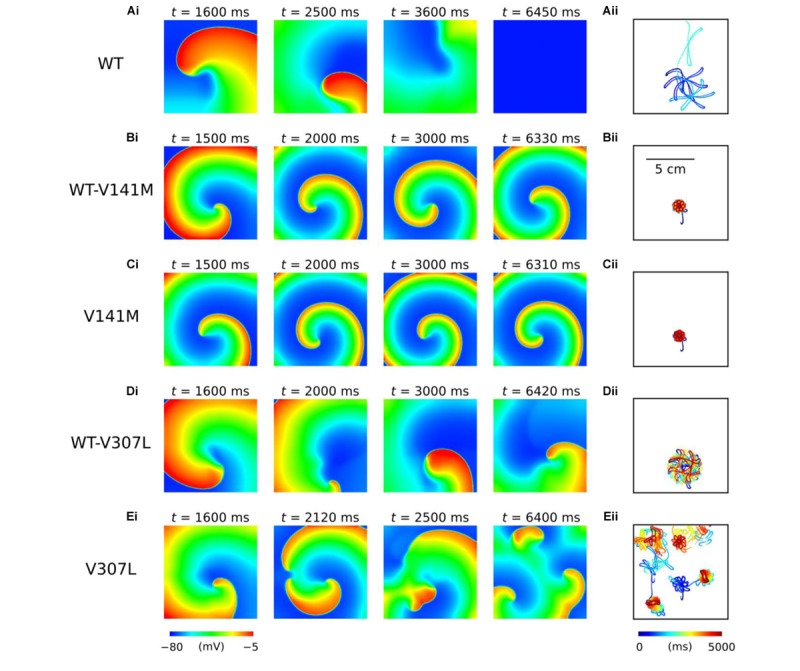
Spiral wave dynamics in 2D human atrial tissue simulations. Snapshots of re-entry are shown at various time points after initiation of a spiral wave in WT **(A)**, WT-V141M **(B)**, V141M **(C)**, WT-V307L **(D)**, and V307L **(E)** conditions **(i)**, with corresponding spiral wave core trajectories **(ii)**. Note that the time at which snapshots were taken differs between mutation conditions.

The functional impact of *KCNQ1*-linked SQT2 mutations on re-entry dynamics was first investigated using an idealised 2D sheet of human atrial tissue. In the WT condition, an S2 stimulus delivered at 259 ms following a train of S1 stimuli with a BCL of 400 ms induced a spiral wave which meandered over a large area for ∼3.7 s before meeting a tissue boundary and terminating (**Figure 5A** and **Supplementary Video S1**). Both heterozygous and homozygous forms of the V141M mutation showed qualitatively similar meandering patterns in tissue. S2 stimuli delivered at 135 ms and 114 ms in WT-V141M and V141M conditions, respectively, produced spiral waves with more stationary trajectories (meandering over a smaller area) which sustained for the complete 5.0 s duration of the simulation (**Figures 5B,C** and **Supplementary Videos S2**, **S3**). In the WT-V307L condition, an S2 stimulus applied at 219 ms produced a spiral wave which also sustained for 5.0 s, meandering with a hypocycloidal trajectory over a considerably larger area than under V141M mutation conditions (**Figure 5D** and **Supplementary Video S4**). In the homozygous V307L mutation condition, however, the S2 stimulus delivered at 201 ms induced a non-stationary and unstable spiral wave which spontaneously degenerated into multiple, regenerative wavelets (**Figure 5E** and **Supplementary Video S5**), resembling AF-like electrical excitations.

### Scroll Wave Dynamics in 3D Anatomical Human Atria Geometry

**FIGURE 6:**
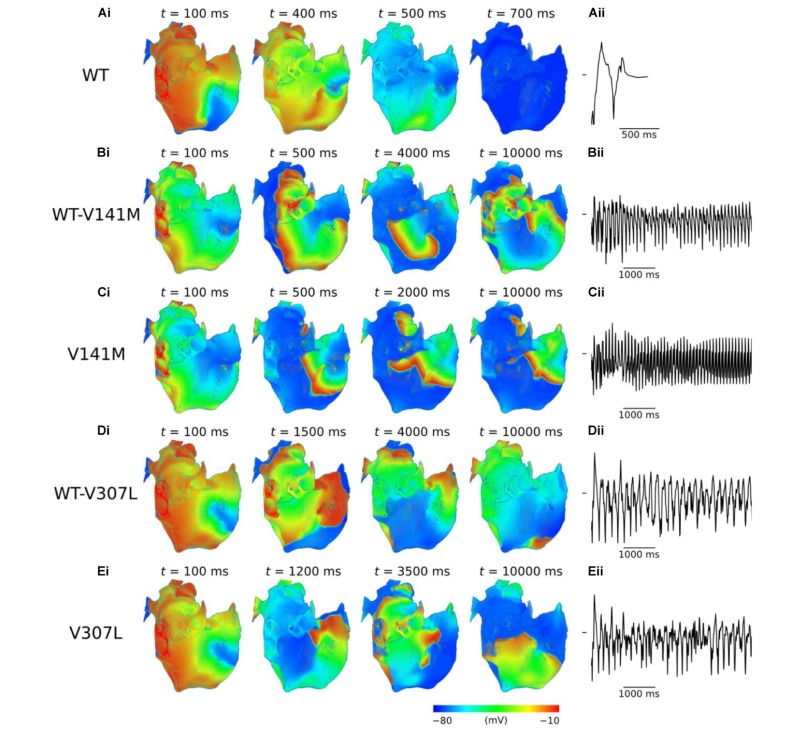
Re-entry dynamics in 3D anatomical human atria. Snapshots of re-entry are shown at various time points after initiation of a scroll wave in WT **(A)**, WT-V141M **(B)**, V141M **(C)**, WT-V307L **(D)**, and V307L **(E)** conditions **(i)**, with corresponding first 5.0 s of pseudo ECG **(ii)**. Note that snapshot times differ between mutation conditions.

Use of the phase distribution method resulted in scroll waves which developed into functional and/or anatomical re-entries in the 3D anatomical human atria model (**Figure 6**). In the WT condition, the initiated scroll wave developed into a transient re-entry, completing two circuits in the RA before self-terminating at ∼0.6 s (**Supplementary Video S6**). This precluded accurate computation of the DF. In the heterozygous WT-V141M mutation condition, the stationary re-entrant wave pattern mirrored that observed in the 2D sheet, with re-entry being driven by a single scroll wave in the RA with a high frequency of rotation (9.1 Hz; **Supplementary Video S7**). In the homozygous V141M mutation condition, re-entry was driven by a single stationary scroll wave in the right atrial appendage (RAA), which had an even higher frequency (10.1 Hz; **Supplementary Video S8**). In both cases the re-entry was stable and persistent (lasting for the full 10.0 s duration of the simulation), and showed no signs of wave break.

In V307L mutation conditions, re-entrant excitation waves meandered to a much larger extent than under the V141M mutation, consistent with observations in the idealised 2D sheet. In the heterozygous WT-V307L condition, the initiated scroll wave developed into an irregular excitation pattern, which eventually anchored to the boundary of the inferior vena cava (IVC) and sustained for 10.0 s (**Supplementary Video S9**). Although the IVC re-entry was persistent, occasional transient micro re-entrant circuits developed around the RAA. In the homozygous V307L condition, the initiated scroll wave moved unpredictably, occasionally breaking and forming multiple wavelets which meandered and collided, as observed in the 2D sheet. This also ultimately degenerated into a persistent circuit around the IVC, sustaining for the full 10.0 s of the simulation (**Supplementary Video S10**). The DF of re-entry under heterozygous and homozygous V307L mutation conditions was 5.3 and 5.8 Hz, respectively.

### Effects of Quinidine on APD, ERP, and Organ-Scale Re-entry Dynamics

As SQTS mutant subunits are expressed heterozygously *in vivo*, the effects of different concentrations of the class Ia anti-arrhythmic drug quinidine were simulated on the cellular APD/ERP and organ scale re-entry dynamics in WT-V141M and WT-V307L mutant conditions only. Quinidine restored the APD to that of the WT level in the WT-V307L but not WT-V141M condition. However, in both mutation conditions quinidine prolonged the ERP in a concentration-dependent manner due to actions on *I*_Na_, especially in the WT-V307L mutation condition in which APD was also prolonged. **Supplementary Table S7** gives a summary of APD and ERP prolongation at 1 and 2 Hz, including that observed in additional simulations in which AF remodelling was considered.

**FIGURE 7:**
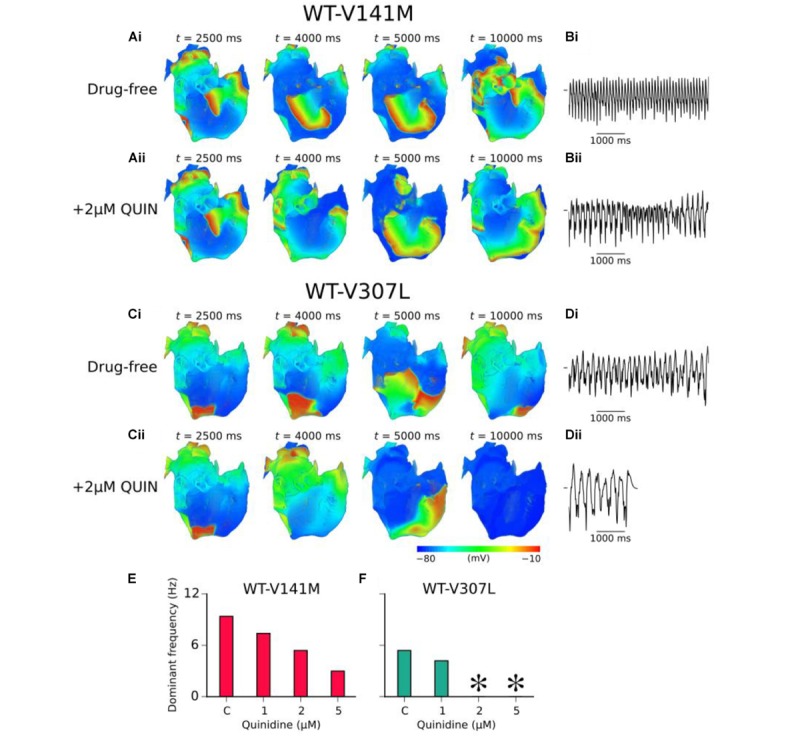
Effects of quinidine on re-entry dynamics under SQT2 mutation conditions. **(A)** Snapshots of re-entrant scroll waves at various time points after initiation of a scroll wave in the 3D anatomical human atria under drug-free WT-V141M **(i)**, and WT-V141M + 2 μM quinidine (QUIN) **(ii)** conditions, with **(B)** corresponding pseudo ECG (pECG) recorded during the final 5.0 s. **(C)** Snapshots of re-entrant scroll waves following re-entry initiation under drug-free WT-V307L **(i)**, and WT-V307L + 2 μM QUIN **(ii)** conditions, with **(D)** corresponding pECGs recorded during the final 5.0 s. Dominant frequencies (DF) calculated from pECGs in control (c) WT-V141M **(E)** and WT-V307L **(F)** conditions and upon application of various concentrations of quinidine. In drug simulations, QUIN was applied at *t* = 2.5 s. ^∗^Denotes DF was not calculated as re-entry termination occurred.

In 3D re-entry simulations, quinidine was applied after 2.5 s under heterozygous SQT2 mutation conditions in which re-entry sustained for 10.0 s in the absence of the drug. Under WT-V141M conditions, all concentrations of quinidine tested failed to terminate re-entry, but decreased its frequency of rotation. Application of both 2 and 5 μM quinidine was sufficient to terminate re-entrant excitations in the WT-V307L condition (termination of re-entry by 2 μM quinidine is shown in **Supplementary Video S11**), reducing the lifespan of re-entrant excitation to 7.5 and 4.0 s, respectively. The mechanism underlying re-entry termination in this condition was increased ERP by quinidine, which reduced the excitable gap for anatomically driven re-entry in the anatomical human atria model. The effects of 2 μM quinidine on re-entry dynamics and the pECG in WT-V141M and WT-V307L conditions are shown in **Figure 7**, as well as the DF for a range of quinidine concentrations. A summary of the effects of quinidine on SQT2-mediated atrial arrhythmias under AF remodelling conditions is shown in **Supplementary Figure S13**.

## Discussion

### Main Findings

The major findings of this study are as follows: (1) The *KCNQ1* V141M but not V307L mutation slowed human SAN pacemaking rate profoundly, through reduced DDR; (2) The V141M and V307L KCNQ1 mutations shortened the human atrial APD through distinct mechanisms – the V141M mutation induced a large instantaneous component of *I*_Ks_ upon membrane depolarisation which shortened the APD, whereas the V307L mutation increased *I*_Ks_ primarily during phase 3 repolarisation, producing more modest APD shortening. The mutations produced opposing effects on the steepness of restitution; (3) Both mutations shortened the tissue excitation wavelength through a reduction in the ERP but not CV across a wide range of pacing rates; (4) In idealised 2D sheets of human atrial tissue, stationary spiral waves were observed with the V141M mutation, whereas spiral waves meandered in the heterozygous V307L mutation condition and wave break occurred in the homozygous V307L condition. In the 3D anatomical human atria model, scroll waves self-terminated in the WT condition, whereas all SQT2 mutation conditions favoured sustenance of re-entry; (5) Quinidine exerted an anti-arrhythmic decrease in the DF of re-entrant excitation in heterozygous forms of the SQT2 mutations, but was effective at terminating scroll waves associated with the V307L mutation only.

### The V141M but not V307L KCNQ1 Mutation Promotes Sinus Bradycardia

In the FS human SAN model, the homozygous form of the V141M mutation abolished pacemaking, whereas the heterozygous form reduced the spontaneous beating rate from 73 bpm in the WT condition to 50 bpm. This is in good agreement with 40–60 bpm (Villafañe et al., 2014) and 50 bpm (Righi et al., 2016) heart rates reported in SQT2 probands with the *KCNQ1* V141M mutation. Upon application of 10 nM ACh, pacemaking rate was further reduced to 22 bpm in the heterozygous V141M mutation condition, and was again abolished altogether in the homozygous V141M mutation condition. Previous computational modelling of the V141M mutation using a rabbit SAN cell model showed abolished pacemaking in a 1:1 WT:mutant heterozygous WT-V141M condition (Hong et al., 2005). However, the present study is the first to reproduce the sinus bradycardia phenotype associated with the V141M mutation using a biophysically detailed model of human SAN electrophysiology (Fabbri et al., 2017). Such alterations to pacemaking activity by the V141M mutation might be expected to impair the ability of the SAN to pace-and-drive the surrounding atrium. In relation to this, Hong et al. (2005) suggested that electrical activity might be initiated in the atrio-ventricular node in such patients, which could potentially explain the lack of apparent P waves.

Heterozygous and homozygous forms of the V307L mutation exerted weaker effects on the SAN pacemaking rate due to less pervasive effects on *I*_Ks_ – the measured rates of 69 and 64 bpm, respectively, were within the normal range of heart rates in humans. It is relevant in this regard that there have been no reports of sinus bradycardia associated with the V307L mutation in *KCNQ1*, with physical examination of the first proband identified revealing no electrophysiological anomalies other than a shortened QT interval, as well as the episode of ventricular fibrillation for which he was admitted to hospital (Bellocq et al., 2004).

### V141M and V307L KCNQ1 Mutations Shorten Human Atrial APD Through Different Mechanisms

The V141M and V307L KCNQ1 mutations have been shown in previous *in silico* studies to shorten the human ventricular APD (Adeniran et al., 2017; Lee et al., 2017). In this study, distinct mechanisms of APD shortening between the mutations in human atrial cells were identified. Whereas the V141M mutation conditions caused a more triangular AP morphology due to increased *I*_Ks_ early during the AP, the V307L mutation conditions preserved the ‘spike and dome’ morphology of the AP due to less pervasive effects on *I*_Ks_ (**Figure 3** and **Supplementary Figure S5**). Furthermore, increased APD dispersion between cells of the pulmonary veins and left atrium under SQT2 mutation conditions was observed (**Supplementary Figure S5**), which may promote high frequency excitation or microreentrant sources around this junction as a mechanism of AF in this context.

Different effects of the V141M and V307L KCNQ1 mutations on the human atrial AP resulted in opposing effects on the maximum slope of APD restitution – additional simulations in **Supplementary Investigation 2.1** revealed that increased *I*_Ks_ conductance alone, similar to kinetic changes seen with the V307L KCNQ1 mutation, increased the slope of restitution, whereas constitutively active *I*_Ks_, as observed for the V141M KCNQ1 mutation, favoured a decrease in the maximum slope of restitution. For the *KCNQ1* V141M mutation, the slope was substantially reduced. This is consistent with the study of Kharche et al. (2012), in which the S140G KCNQ1 mutation which includes an instantaneous component of *I*_Ks_ similar to that induced by the V141M mutation, was shown to reduce markedly the maximum slope of restitution in the CRN human atrial cell model (Courtemanche et al., 1998). The V307L mutation, on the other hand, was shown to increase the slope of restitution, which was also reported in the investigation of the *KCNQ1* V307L mutation in human ventricles by Adeniran et al. (2017). Both of these key findings were reproduced in supplementary simulations using the human atrial model of Grandi et al. (2011) (**Supplementary Investigation 2.2**).

### SQT2 Mutations Promote Human Atrial Arrhythmogenesis

Action potential duration shortening at the cellular level manifested as a reduction in the ERP across all pacing rates for both SQT2 mutations. The CV was largely unaffected, and thus the profound reduction in the excitation WL observed under SQT2 mutation conditions can be attributed almost exclusively to the reduction in ERP. This is in contrast to findings pertaining to *KCNJ2*-linked SQTS presented in our previous study (Whittaker et al., 2017b), where reduced WL was found to be mediated by a decrease in both ERP and CV; this emphasises the value of multi-scale computational modelling in elucidating phenotypic differences between different variants of the SQTS. In idealised 2D sheet simulations, WT tissue did not support a sustained spiral wave, whereas the V141M and V307L mutations both favoured sustenance of re-entry. The V141M mutation produced stationary spiral waves, which were also observed in the study of Kharche et al. (2012) for the S140G KCNQ1 mutation which similarly induces an instantaneous component of *I*_Ks_ (Chen et al., 2003). In contrast, the V307L mutation conditions produced spiral waves which meandered to a larger extent, spontaneously breaking and forming multiple wavelets in the homozygous condition. This mutant form showed a steep (>1) maximum slope of restitution and alternans, which are known to promote electrical instability in cardiac tissue.

In 3D human atria simulations the V141M mutation produced stationary scroll waves, even in the presence of electrical and anatomical heterogeneities. The persistent nature of re-entry in this condition compared to the WT, in which scroll waves quickly self-terminated, can be explained in terms of tissue excitation WL. Increased *I*_Ks_ associated with the V141M mutation abbreviated APD and ERP, which consequently reduced the excitation WL. This is a measure of the spatial requirement for a functional re-entrant circuit, and thus reduced WL facilitates conduction of high rate excitation waves within a limited atrial mass (Kharche et al., 2012; Whittaker et al., 2017b). The findings of this study thus substantiate a causative link between the *KCNQ1* V141M mutation and multiple reports of recalcitrant AF in affected patients (Hong et al., 2005; Villafañe et al., 2014; Righi et al., 2016). There have been no reported episodes of AF consequent to the V307L mutation to date. However, as the number of V307L SQTS patients is very small (Hu et al., 2017), and AF can be paroxysmal and/or asymptomatic, atrial arrhythmias arising from this mutation cannot be ruled out. The phenotypically accurate computational models in this study predicted that the V307L KCNQ1 mutation facilitates sustenance of re-entrant excitations in the human atria, albeit with decreased stability, stationarity, and DF compared to V141M mutation conditions.

### Quinidine Controls Rate but not Rhythm of Arrhythmic Atrial Excitations in SQT2

To date, no specific blockers of *I*_Ks_ are in clinical use (Hancox et al., 2018). The current front-line pharmacological treatment for *hERG*-related SQTS patients is the class Ia anti-arrhythmic drug quinidine (Gaita et al., 2004; Hu et al., 2017) – a multi-channel blocker which exerts a mild blocking effect on *I*_Ks_. Under the WT-V141M mutation condition, quinidine reduced the DF of re-entry, but did not terminate re-entrant activity at any of the concentrations tested. This is consistent with the findings of Righi et al. (2016) who reported that recurrent AF associated with the *KCNQ1* V141M mutation was unresponsive to multiple anti-arrhythmic agents (failing to revert to sinus rhythm), including (hydro)quinidine (Righi et al., 2016). Re-entrant excitations in the human atria under the WT-V307L mutation condition, on the other hand, were responsive to quinidine therapy, with concentrations of 2 and 5 μM terminating re-entry in our model. The findings of the 3D simulations were consistent with cellular level APD/ERP predictions, in which quinidine was shown to effectively restore the APD only in the WT-V307L condition, but consistently increased the ERP in a dose-dependent manner under both mutation conditions (including in the presence of AF remodelling; **Supplementary Table S7**). These results suggest that in the setting of SQT2-mediated atrial arrhythmias, quinidine may be a more effective strategy for rate control than rhythm control.

It has been reported previously that quinidine was ineffective at prolonging the QT interval in the setting of *KCNQ1* V141M-mediated SQT2 (Righi et al., 2016). As SQT2 is caused by a gain-of-function in *I*_Ks_, it is likely that an anti-arrhythmic drug which blocks *I*_Ks_ to a larger degree is required to reverse the phenotype. In a previous computational study of SQT2 in the human ventricles by Adeniran et al. (2017), *I*_Ks_ block was demonstrated to effectively terminate re-entrant excitations associated with the V307L mutation in *KCNQ1*, as well as to restore the APD/QT interval in both V307L and V141M conditions. Selective *I*_Ks_ block as a potential therapeutic strategy has also been supported in an experimental study (Campbell et al., 2013). *I*_K1_ has also been suggested to be a potential therapeutic target in the context of V141M-mediated SQT2, based on the reduction in transmural APD heterogeneity and prolongation of the QT interval observed following *I*_K1_ block in computational models of the human ventricles (Lee et al., 2017).

### Limitations

There are a number of limitations associated with the simulations presented in this study. Potential limitations of the 3D anatomical human atria, regional cell models, and drug binding models have been discussed in our previous publications (Colman et al., 2013; Ni et al., 2017; Whittaker et al., 2017a,b). Specific limitations to be considered here are as follows. (1) Previous *in vitro* experiments have shown that blocking potency of *I*_Ks_ blockers can be reduced for recombinant channels containing *KCNQ1*-linked SQT2 mutations, as observed for chromanol 293B (Lerche et al., 2007; El Harchi et al., 2010) but not mefloquine (El Harchi et al., 2010). Whether or not quinidine block of *I*_Ks_ is altered by the SQT2 mutations was not considered due to lack of experimental data, although even at the highest concentration tested (5 μM), the block of *I*_Ks_ was small (∼10%). (2) Late sodium current, *I*_NaL_, is not present in the CNZ model. Quinidine block of *I*_NaL_ could potentially reduce the APD prolongation observed in this study, although the presence and contribution of *I*_NaL_ in human atrial myocytes at physiological temperature remains to be confirmed (Poulet et al., 2015). (3) There are currently no data on the presence or absence of electrical and/or structural remodelling in the SQTS. Inclusion of these factors was considered in the **Supplementary Material** for completeness – these additional simulations do not change the fundamental mechanistic conclusions drawn in this study. (4) The heterozygous formulations of SQT2 mutants used in this study relied on the simplifying assumption that both V141M and V307L *I*_Ks_ behave similarly to a 50:50 mixture of WT and mutant channels. In reality, the channel populations may be more complex, with each channel consisting of both WT and mutant *KCNQ1* subunits. In our previous study (Zhang et al., 2008), the effects of varying mutant subunit composition in SQT2 were investigated, based on different expression/co-expression ratios used by Bellocq et al. (2004) in the original paper describing the V307L KCNQ1 mutation. In that study (Zhang et al., 2008), it was found that the degree of shortening of the ventricular APD and QT interval increased progressively with the level of V307L expression. Furthermore, in our previous study (Adeniran et al., 2017), the heterozygote formulation used for V307L was shown to reproduce QT interval shortening and increased T wave associated with the SQTS phenotype, thus supporting the approach adopted in this study. (5) The effects of human atrial contraction were not considered, which could feasibly modulate the atrial AP under SQTS conditions (Whittaker et al., 2015), and thus re-entrant electrical activity. (6) Scroll wave dynamics in the 3D anatomical human atria model should be interpreted with caution, as dynamic behaviour of re-entrant excitations, including wave break, can depend critically on initial conditions, especially in such a complex geometry (Benson et al., 2007).

## Conclusion

The multi-scale computational approach adopted in this study allowed phenotypic differences associated with two distinct *KCNQ1*-linked SQTS mutations to be assessed. Furthermore, the response of arrhythmic excitation waves to clinically relevant doses of quinidine under SQT2 conditions was probed. The simulations substantiated a causative link between the *KCNQ1* V141M mutation and an AF/sinus bradycardia phenotype which has been observed clinically. In addition, the V307L mutation in *KCNQ1* was predicted to promote human atrial arrhythmogenesis whilst not significantly affecting pacemaking function. Quinidine was shown to be useful for rate control of atrial arrhythmias associated with SQT2, but appears likely to be less reliable for rhythm control in this setting.

## Author Contributions

DW, JH, and HZ conceived the experiments. DW developed and validated the computer models and performed the numerical experiments and analysis. DW, MC, and HN provided the computing tools. All authors wrote the manuscript.

## Conflict of Interest Statement

The authors declare that the research was conducted in the absence of any commercial or financial relationships that could be construed as a potential conflict of interest.
