# Supplementary material for: Human Atrial Arrhythmogenesis and Sinus Bradycardia in KCNQ1-Linked Short QT Syndrome: Insights From Computational Modelling
Source: Front Physiol. 2018 Oct 4;9:1402. doi: 10.3389/fphys.2018.01402 (PMC6180159; doi:10.3389/fphys.2018.01402)
Supplement: Supplementary file 12 [file Data_Sheet_1.pdf]

Supplementary Material

Human atrial arrhythmogenesis and sinus bradycardia in *KCNQ1*-linked short QT syndrome: insights from computational modelling

Dominic G. Whittaker, Michael A. Colman, Haibo Ni, Jules C. Hancox*, Henggui Zhang*

*** Correspondence:** Corresponding Author(s):
jules.hancox@bristol.ac.uk (JH); henggui.zhang@manchester.ac.uk (HZ)

1. Supplementary Methods

1.1. Markov chain models of WT and SQT2 mutant I_{Ks}

The Markov chain (MC) scheme used in this study, which is based on the work of Silva and Rudy using expressed human I_{Ks} data at physiological temperature (Silva and Rudy, 2005), is shown in Figure S1. The MC scheme is based on a two-stage voltage sensor transition principle, described in the work of Zagotta *et al*. on Shaker K+ channels (Shaker potassium channel gating. II: Transitions in the activation pathway, 1994, Shaker potassium channel gating. III: Evaluation of kinetic models for activation, 1994), and thus accounts for four identical subunits which undergo two conformational transitions to reach the activated state. The multiple closed-state transitions before channel opening reproduce the sigmoidal activation of I_{Ks}, and two open states best reproduce slow deactivation (Silva and Rudy, 2005). The equations for I_{Ks} are

$$I_{\mathrm{Ks}} = g_{\mathrm{Ks}}(O_1 + O_2)(V - E_{\mathrm{Ks}}), \qquad \text{(S1)}$$

$$\frac{\mathrm{d}C_1}{\mathrm{d}t} = \beta C_2 - 4\alpha C_1, \qquad \text{(S2)}$$

$$\frac{\mathrm{d}C_2}{\mathrm{d}t} = 2\beta C_3 + \delta C_6 + 4\alpha C_1 - (3\alpha + \gamma + \beta)C_2, \qquad \text{(S3)}$$

$$\frac{\mathrm{d}C_3}{\mathrm{d}t} = 3\beta C_4 + \delta C_7 + 3\alpha C_2 - (2\alpha + 2\gamma + 2\beta)C_3, \qquad \text{(S4)}$$

$$\frac{\mathrm{d}C_4}{\mathrm{d}t} = 4\beta C_5 + \delta C_8 + 2\alpha C_3 - (\alpha + 3\gamma + 3\beta)C_4, \qquad \text{(S5)}$$

$$\frac{\mathrm{d}C_5}{\mathrm{d}t} = \delta C_9 + \alpha C_4 - (4\gamma + 4\beta)C_5, \qquad \text{(S6)}$$

$$\frac{\mathrm{d}C_6}{\mathrm{d}t} = 2\beta C_7 + \gamma C_2 - (3\alpha + \delta)C_6, \qquad \text{(S7)}$$

$$\frac{\mathrm{d}C_7}{\mathrm{d}t} = 3\beta C_8 + 2\delta C_{10} + 3\alpha C_6 + 2\gamma C_3 - (2\alpha + \gamma + 2\beta + \delta)C_7, \qquad \text{(S8)}$$

$$\frac{\mathrm{d}C_8}{\mathrm{d}t} = 4\beta C_9 + 2\delta C_{11} + 2\alpha C_7 + 3\gamma C_4 - (\alpha + 2\gamma + 3\beta + \delta)C_8, \qquad \text{(S9)}$$

$$\frac{\mathrm{d}C_9}{\mathrm{d}t} = 2\delta C_{12} + \alpha C_8 + 4\gamma C_5 - (3\gamma + 4\beta + \delta)C_9, \quad \text{(S10)}$$

$$\frac{\mathrm{d}C_{10}}{\mathrm{d}t} = 3\beta C_{11} + \gamma C_7 - (2\alpha + 2\delta)C_{10}, \quad \text{(S11)}$$

$$\frac{\mathrm{d}C_{11}}{\mathrm{d}t} = 4\beta C_{12} + 3\delta C_{13} + 2\alpha C_{10} + 2\gamma C_8 - (\alpha + \gamma + 3\beta + 2\delta)C_{11}, \quad \text{(S12)}$$

$$\frac{\mathrm{d}C_{12}}{\mathrm{d}t} = 3\delta C_{14} + \alpha C_{11} + 3\gamma C_9 - (2\gamma + 4\beta + 2\delta)C_{12}, \quad \text{(S13)}$$

$$\frac{\mathrm{d}C_{13}}{\mathrm{d}t} = 4\beta C_{14} + \gamma C_{11} - (\alpha + 3\delta)C_{13}, \quad \text{(S14)}$$

$$\frac{\mathrm{d}C_{14}}{\mathrm{d}t} = 4\delta C_{15} + \alpha C_{13} + 2\gamma C_{12} - (\gamma + 4\beta + 3\delta)C_{14}, \quad \text{(S15)}$$

$$\frac{\mathrm{d}C_{15}}{\mathrm{d}t} = \eta O_1 + \gamma C_{14} - (\theta + 4\delta)C_{15}, \quad \text{(S16)}$$

$$\frac{\mathrm{d}O_1}{\mathrm{d}t} = \omega O_2 + \theta C_{15} - (\psi + \eta)O_1, \quad \text{(S17)}$$

$$\frac{\mathrm{d}O_2}{\mathrm{d}t} = \psi O_1 - \omega O_2, \quad \text{(S18)}$$

where g_{Ks} is the maximal channel conductance, V is the membrane potential, E_{Ks} is the reversal potential, O_1 and O_2 are open states, C_1-C_{15} are closed states, and α, β, δ, γ, η, θ, ω, and ψ are transition rates. The reversal potential is given by

$$E_{\mathrm{Ks}} = \frac{R \cdot T}{F} \ln \frac{[K_\mathrm{o}^+] + P_{\mathrm{Na/K}} \cdot [Na_\mathrm{o}^+]}{[K_\mathrm{i}^+] + P_{\mathrm{Na/K}} \cdot [Na_\mathrm{i}^+]}, \quad \text{(S19)}$$

where R is the molar gas constant, T is the temperature, F is the Faraday constant, o and i subscripts for concentrations denote extracellular and intracellular, respectively, and $P_{\mathrm{Na/K}}$ is ratio of Na^+:K^+ permeability (set to 0.01833 (Silva and Rudy, 2005)).

Parameters describing *KCNQ1* V307L mutant and wild type (WT) I_{Ks} were incorporated exactly as described previously (Adeniran et al., 2017). The response of WT and mutant currents to simulated voltage clamps and corresponding *I-V* relation and steady state activation is shown in Figures S2A,B. Furthermore, as this study focuses on I_{Ks} in the human atria, the responses of the MC models of WT and V307L mutant I_{Ks} to AP clamps using a (simulated) human atrial AP waveform were compared with experimental data (El Harchi et al., 2010). The resulting I_{Ks} profiles are shown in Figure S2C, where close concordance can be seen with experiment.

The MC formulation of I_{Ks} was subsequently employed to develop a model of the V141M mutation in *KCNQ1*, first described by Hong *et al*. (Hong et al., 2005). An additional parameter, ξ, was introduced to account for the constitutively active, voltage-independent component of I_{Ks} observed in *KCNQ1* V141M mutant channels (Hong et al., 2005; Restier et al., 2008). In the V141M mutation condition, the formulation for I_{Ks} was thus modified to

$$I_{\mathrm{Ks}} = g_{\mathrm{Ks}}(O_1 + O_2 + \xi)(V - E_{\mathrm{Ks}}), \quad \text{(S20)}$$

where the MC state equations were the same as given previously. The response of V141M mutant currents to simulated voltage clamps and corresponding *I-V* relation and steady state activation is shown in Manuscript Figure 1. It should be noted that the trace for WT I_{Ks} in Manuscript Figure 1 is not identical to that presented in Figure S2 due to differences in both the protocol used and a large difference in temperature (given in figure legends). Transition rates for WT, V307L, and V141M mutant channels (corresponding to the MC scheme in Figure S1) are given in Figure S3.

1.2. Modelling electrophysiology of the human atria

An updated version of the Colman *et al.* model of the human atrial AP, which is well suited to the simulation of re-entrant atrial arrhythmias (Colman et al., 2013, 2017; Whittaker et al., 2017b), was used for the modelling in this study (Ni et al., 2017) (and is referred to as the CNZ model). The I_{Ks} equations (native to the parent CRN model (Courtemanche et al., 1998)) were replaced with the WT formulation of I_{Ks}, with a conductance of 0.161 nS/pF (which places the current density within the range observed in human atrial myocytes (Caballero et al., 2010) – Supplementary Figure S4). Furthermore, the MC model of I_{Kr}/hERG developed in our previous study (Whittaker et al., 2017a) was incorporated into the CNZ model with the conductance set to $0.0111 \cdot [K^+]_o^{0.59}$, where $[K^+]_o$ is the extracellular potassium concentration (allowing for simulation of drug binding of quinidine to I_{Kr} – see Supplementary Methods 1.5). The resulting WT AP had an APD_{90} of 250.0 ms at 1 Hz, which is well within the experimentally-measured range of APDs in human atrial myocytes (Bosch et al., 1999; Calum J Redpath, 2006; Dobrev and Ravens, 2003; Katoh et al., 2005; Kim et al., 2002; Pau et al., 2007) – see Table S1. This ensured that the role of I_{Kr} on the AP remained the same as in the original Colman *et al.* model, based on the original data from human atrial myocytes (Courtemanche et al., 1998; Wang et al., 1994).

A family of regional cell models was developed based on experimentally-measured changes in maximal current density of several ionic currents in different regions of the atria (Colman et al., 2013), where conductance changes were implemented relative to the baseline right atrium (RA) model. Data from canine atrial myocytes were used primarily (Burashnikov et al., 2004; Cha et al., 2005; Datino et al., 2010; Ehrlich et al., 2003; Feng et al., 1998; Li et al., 2001), as these are much more readily available than human atrial data (Caballero et al., 2010; Gong et al., 2008; Katoh et al., 2005). Changes to maximal ionic conductances used to create a family of regional cell models can be seen in Table S2. The response of regional cell models to SQT2 mutants at 1 Hz is shown in Figure S5.

APD restitution curves were measured using an S1-S2 protocol. 400 conditioning S1 stimuli were delivered at a pacing frequency of 1 Hz (stimulus strength 20 pA/pF and duration 2.0 ms), following which an S2 stimulus was applied at varying intervals. APD restitution curves were generated by plotting the APD (measured from the maximum upstroke velocity to the point at which the membrane potential crossed −70 mV) against the corresponding diastolic interval (DI), which was computed using DI = S2 – APD, where S2 denotes the S1-S2 interval. Maximum slope of restitution was determined as the maximal derivate of APD against DI.

1.3. Modelling the effects of AF-induced remodelling

In this study, as a theoretical consideration, the effects of AF-induced electrical remodelling were simulated using the same approach adopted in previous studies (Colman et al., 2013, 2017). Evidence of AF-induced remodelling in the SQTS is currently lacking; nevertheless, it is useful to consider electrical remodelling which could potentially arise from persistent forms of AF induced by genetic mutations in the SQTS. Consistent with experimental evidence (Bosch et al., 1999; Wagoner et al.,

1999; Workman et al., 2001), AF-induced remodelling of atrial myocytes was assumed to markedly reduce the maximal conductance of I_{CaL}, I_{to}, and I_{Kur}, whereas the conductance of I_{K1}, I_{Ks}, and I_{NaCa} were all increased. A list of parameter changes in the AF model relative to the baseline sinus rhythm (SR) model is given in Table S3. In addition, in tissue simulations with AF-induced electrical remodelling, the value of the diffusion coefficient, **D**, was reduced by 40% in order to simulate possible connexin and structural remodelling (a reduction in **D** increases the effective tissue size) associated with AF (Colman et al., 2013, 2017). As structural/connexin remodelling may occur without electrical remodelling, simulations were also performed with **D** reduced by 40% but no AF-induced electrical remodelling.

A comparison of the CNZ model AP under SR and AF remodelling conditions at 1 Hz is shown and compared to experimentally-measured human atrial APs from Dobrev *et al.* (Dobrev and Ravens, 2003), Bosch *et al.* (Bosch et al., 1999), Workman *et al.* (Workman et al., 2001), van Wagoner *et al.* (Van Wagoner and Nerbonne, 2000), and Voigt *et al.* (Voigt et al., 2012) in Figure S6. The simulated AF conditions produced changes to the AP which were strongly concordant with those observed experimentally; namely, (i) the markedly shortened APD associated with AF-induced electrical remodelling, and (ii) the more triangular AP morphology. In SQT2 mutation conditions, the simplifying assumption was made that electrical remodelling associated with AF was identical to that in the absence of SQTS gene mutations (minus effects on I_{Ks}), and was thus also implemented using the parameters given in Table S3. It should also be noted that whereas remodelling of I_{Kur} under AF conditions was included in the model based on certain experimental data (Caballero et al., 2010; Christ et al., 2004; Wagoner et al., 1997), other studies have reported no changes in I_{Kur} under human AF remodelling conditions (not shown in Table S3) (Bosch et al., 1999; Workman et al., 2001). Reduced I_{Kur} was included in this study as it better reproduced the AP morphology associated with AF remodelling.

1.4. Modelling electrophysiology of the human sinoatrial node

The recently-developed human sinoatrial node (SAN) model of Fabbri *et al.* (Fabbri et al., 2017), referred to as the FS model, was used to simulate primary pacemaking in the human heart in this study. The native equations for I_{Ks} were replaced with the MC formulation developed in this study, with the maximal conductance set to a value of 0.00965 nS/pF. This gave a natural cycle length of 818 ms, which is in good agreement with the value of 828 ± 21 ms measured experimentally by Verkerk *et al.* (Verkerk et al., 2007). It should be noted that this involved updating the reversal potential, E_{Ks}, from the potentially unphysiological value of −49 mV in the original model, to the more realistic value of ~−75 mV for consistency with CNZ human atrial simulations. This meant that the new formulation of I_{Ks} in the updated FS model is always an outward current, and thus current density had to be reduced in order to give the same contribution from I_{Ks} as in the original model. Furthermore, it also meant that I_{Ks} always shortens the APD as it is an outward current exclusively, so the APD_{50} and APD_{90} values are smaller than published values from the original FS model (Fabbri et al., 2017).

The effects of autonomic modulation by 10 nM acetylcholine (ACh) in the FS model were simulated through a −7.5 mV shift in funny current (I_f) activation, a 3.1% reduction in L-type calcium channel current (I_{CaL}) maximum conductance, and a 7% decrease in sarcoplasmic reticulum Ca^{2+} uptake (in addition to the effects of the acetylcholine-activated potassium current, $I_{K,ACh}$) (Fabbri et al., 2017). A comparison between FS model AP characteristics (in the absence of autonomic modulation) and experimentally-measured values is shown in Figure S7. The diastolic depolaristion rate (DDR) was

calculated using DDR = (TOP−MDP)/(t_{TOP}−t_{MDP}), where TOP is the take-off (threshold) potential, MDP is the maximum diastolic potential (i.e. the minimum potential attained), and t_{TOP} and t_{MDP} are the times at which the TOP and MDP occur, respectively.

1.5. Modelling pharmacological actions of quinidine

In this study, the actions of quinidine on human atrial electrophysiology were represented using the same formulations and IC_{50} (half maximal inhibitory concentration) values presented in our previous study (Whittaker et al., 2017a). Briefly, in that study, state-dependent models of the actions of quinidine were developed for I_{Na} and I_{Kr}, and a simple pore block approach was used to describe block of I_{CaL}, I_{to}, I_{Ks}, I_{K1}, and I_{NaL} (late sodium current block was not included in the present study). Quinidine block of I_{Kur} was additionally accounted for in the present study (Nenov et al., 1998). In the simple pore block theory (Brennan et al., 2009), the maximal conductance g_i of an ionic current type i is modified in a concentration-dependent manner, such that

$$g_i = g_{\mathrm{control},i} \frac{1}{1+([\mathrm{IC}_{50}]_i/[D])^{nH}}, \qquad \text{(S21)}$$

where $g_{\mathrm{control},i}$ represents the maximal conductance of the i channel in drug-free conditions, $[D]$ is the concentration of the drug, and nH is the Hill coefficient. A summary of IC_{50} values for quinidine block of multiple ionic currents is given in Table S4.

As described previously (Whittaker et al., 2017a), quinidine was assumed to bind to activated, inactivated, and rested sodium channels. The formulation for I_{Na} is thus given by the guarded receptor equations

$$I_{\mathrm{Na}} = g_{\mathrm{Na}}(1 - b_{\mathrm{A}} - b_{\mathrm{I}} - b_{\mathrm{R}})m^3hj(V - E_{\mathrm{Na}}), \qquad \text{(S22)}$$

$$\frac{\mathrm{d}b_{\mathrm{A}}}{\mathrm{d}t} = k_{\mathrm{A}}[D]m^3hj(1 - b_{\mathrm{A}} - b_{\mathrm{I}} - b_{\mathrm{R}}) - l_{\mathrm{A}}b_{\mathrm{A}}, \qquad \text{(S23)}$$

$$\frac{\mathrm{d}b_{\mathrm{I}}}{\mathrm{d}t} = k_{\mathrm{I}}[D](1 - hj)(1 - b_{\mathrm{A}} - b_{\mathrm{I}} - b_{\mathrm{R}}) - l_{\mathrm{I}}b_{\mathrm{I}}, \qquad \text{(S24)}$$

$$\frac{\mathrm{d}b_{\mathrm{R}}}{\mathrm{d}t} = k_{\mathrm{R}}[D](1 - m^3)hj(1 - b_{\mathrm{A}} - b_{\mathrm{I}} - b_{\mathrm{R}}) - l_{\mathrm{R}}b_{\mathrm{R}}, \qquad \text{(S25)}$$

where g_{Na} is the maximal channel conductance, b_A, b_I, and b_R are the fractional blocks of activated, inactivated, and resting states, respectively, m is the activation gate, h and j are the fast and slow inactivation gates of the sodium channel, respectively, E_{Na} is the Na^+ reversal potential, and all other parameters retain their previous definitions. Binding and unbinding rates of quinidine to sodium channels are given in Table S5, and model fits to experimental data (Koumi et al., 1992) are shown in Figure S8.

Quinidine was assumed to block the open and inactivated states of hERG channels (Whittaker et al., 2017a), which was modelled through addition of drug-bound open and inactivated states to the MC model of I_{Kr}/hERG used in this study. The formulation for I_{Kr} is given by

$$I_{\mathrm{Kr}} = g_{\mathrm{Kr}}O(V - E_{\mathrm{Kr}}), \qquad \text{(S26)}$$

$$\frac{\mathrm{d}C1}{\mathrm{d}t} = \beta C2 - \alpha C1, \qquad \text{(S27)}$$

$$\frac{\mathrm{d}C2}{\mathrm{d}t} = \alpha C1 + \beta_1 C3 - (\beta + \alpha_1)C2, \quad \text{(S28)}$$

$$\frac{\mathrm{d}C3}{\mathrm{d}t} = \alpha_1 C2 + \beta_2 O + \mu I - (\beta_1 + 2\alpha_2)C3, \quad \text{(S29)}$$

$$\frac{\mathrm{d}I}{\mathrm{d}t} = \alpha_2 C3 + \beta_i O + l_I I^* - (\mu + \alpha_i + k_I)I, \quad \text{(S30)}$$

$$\frac{\mathrm{d}O}{\mathrm{d}t} = \alpha_2 C3 + \alpha_i I + l_A O^* - (\beta_2 + \beta_i + k_A)O, \quad \text{(S31)}$$

$$\frac{\mathrm{d}I^*}{\mathrm{d}t} = k_I[D]I - l_I I^*, \quad \text{(S32)}$$

$$\frac{\mathrm{d}O^*}{\mathrm{d}t} = k_A[D]O - l_A O^*, \quad \text{(S33)}$$

where g_{Kr} is the maximal channel conductance, O an open state, I an inactivated state, *C1*, *C2*, and *C3* are closed states, O^* and I^* represent drug-bound open and inactivated states, respectively, E_{Kr} is the K^+ reversal potential, and k_{X} and l_{X} are binding and unbinding rate constants for states of type X, respectively. Binding and unbinding rates of quinidine to hERG channels are given in Table S6, and model fits to experimental data (McPate et al., 2008; Paul et al., 2002) are shown in Figure S9. Full details of the parameter estimation procedure for drug binding models of quinidine are given in our previous study (Whittaker et al., 2017a).

1.6. Tissue simulations

Propagation of excitation waves in tissue was described using the monodomain equation (Clayton et al., 2011),

$$\frac{\partial V}{\partial t} = \nabla(\mathbf{D}\nabla V) - \frac{I_{\mathrm{ion}}}{C_{\mathrm{m}}}, \quad \text{(S34)}$$

where V is the transmembrane voltage, **D** is the tensor of diffusion coefficients, I_{ion} is the total ionic current, and C_{m} is the membrane capacitance. Eq. (S34) was solved using a finite difference PDE solver based on the explicit forward Euler method and Strang splitting scheme (Ni et al., 2017). The pseudo-ECG (pECG) was calculated according to (Plonsey and Barr, 2013), i.e.

$$\Phi(x', y', z') = \int (-\nabla V) \cdot \left[\nabla \frac{1}{r}\right] \mathrm{d}\Omega, \quad \text{(S35)}$$

$$r = [(x - x')^2 + (y - y')^2 + (z - z')^2]^{1/2}, \quad \text{(S36)}$$

where Φ is a unipolar potential generated by the multicellular tissue preparation, r is the distance between a source point (x, y, z) and the coordinate of a virtual electrode (x', y', z'), and Ω is the domain of integration (i.e. atrial tissue volume).

1.7. One-dimensional (1D) tissue models

An electrically-homogeneous, isotropic right atrial 1D strand of 100 nodes with spacing 0.25 mm was used to assess the effects of *KCNQ1* mutations on the effective refractory period (ERP), conduction velocity (CV), and wavelength of re-entry (WL) (Whittaker et al., 2017b). Steady state

variables from single cell models across a range of basic cycle lengths (BCL) at intervals of 10 ms were saved and read into the 1D model as initial conditions for computation of steady-state restitution curves for ERP, CV, and WL. When measuring the ERP, a threshold of −20 mV was used to define atrial activation (Whittaker et al., 2017b). The CV was determined by computing the quotient of the distance of the centre section of the 1D strand and the activation time of the same section by an excitation wave. The simulated CV in the 1D strand under baseline conditions was 0.7 ms^{-1}, close to experimental measurements in the RA free wall (Fedorov et al., 2010). Tissue WL was calculated according to WL = CV × ERP.

1.8. Two-dimensional (2D) tissue models

In order to characterise spiral wave dynamics in WT and SQT2 mutant tissue, an isotropic 2D sheet of electrically-homogeneous atrial tissue with dimensions 100×100 mm^2 and spatial step 0.25 mm was employed (Whittaker et al., 2017b). Spiral waves were initiated using a cross-field S1-S2 stimulation protocol, wherein one edge of the sheet was paced with four conditioning S1 stimuli at a BCL of 400 ms using steady-state initial conditions obtained in a single cell environment, followed by application of an S2 stimulus of area 50×50 mm^2 in the lower left quadrant of the sheet (Whittaker et al., 2017b). The S2 stimulus was applied at time, t = ERP+20 ms, which caused a uni-directional conduction block and resulted in development of a spiral wave under all conditions investigated. The resulting behaviour over a 5.0 s simulation period was recorded. The cores of spiral waves were tracked using the method of identifying phase singularities with time-delay embedding of the transmembrane potential (Bray and Wikswo, 2002) (using a time delay of τ = 10.0 ms).

1.9. Heterogeneous three-dimensional (3D) anatomical human atria geometry

The behaviour of re-entrant excitation waves in an anatomically-realistic setting was determined using a 3D human atria structured grid geometry based on the Visible Female dataset (Seemann et al., 2006), as has been employed in several of our previous studies (Colman et al., 2013, 2017; Kharche et al., 2012; Whittaker et al., 2017b). Fibre orientations were incorporated using a semi-automatic rule-based approach (Krueger et al., 2011, 2013), and the 3D geometry was segmented into the same distinct regions of the human atria as described in (Colman et al., 2013; Whittaker et al., 2017b). The transverse value of the diffusion coefficient, **D**, was set to 0.1 mm^{2}ms^{-1}, where an anisotropy ratio of 3:1 was applied in directions longitudinal and transverse to fibres in the atrial working myocardium, and a ratio of 9:1 along the fast conducting bundles of the Bachmann’s bundle, crista terminalis, and pectinate muscles (Colman et al., 2013; Whittaker et al., 2017b). The spacing between nodes, Δx, was set to Δx = 0.33 mm as determined by the data set resolution (Seemann et al., 2006). The 3D anatomical human atria geometry segmentation and fibre orientations are shown in Figure S10, as well as measured atrial activation times, which were in close agreement with experimental measurements (Lemery et al., 2007). Following stimulation of the SAN region (modelled electrically as CT tissue for simplicity (Colman et al., 2013, 2017)), the excitation spread radially outwards, first entering the LA at 29 ms (cf. 31±13 ms (Lemery et al., 2007)), fully activating the RA after 91 ms (cf. 93±17 ms (Lemery et al., 2007)), and with a latest LA activation time of 107 ms (cf. 116±118 ms (Lemery et al., 2007)).

In this study, re-entrant scroll waves were initiated using the phase distribution method (Biktashev and Holden, 1998) – for detailed descriptions and illustrations of the phase distribution method see (Biktashev and Holden, 1998; Colman et al., 2017; Whittaker et al., 2017b). Where sustained re-entrant activity was initiated, a power spectrum was obtained through Fourier transform analysis of pECG time series (the virtual electrode was placed approximately in the centre of the RA cavity). The dominant frequency (DF) was computed using Matlab based on the largest peak in the power

spectrum density (Whittaker et al., 2017b). It should be noted that this simple approach to computing the DF, described in our previous studies (Colman et al., 2013; Whittaker et al., 2017b), is different from the more commonly used definition (e.g. see Ng *et al*. (Ng et al., 2006)). For simulating the effects of quinidine on re-entrant wave patterns, state variables for each node within the 3D anatomical model were saved after 2.5 s of a 10.0 s episode of sustained re-entrant activity in drug-free conditions. These were then used as initial conditions for new 3D simulations of duration 7.5 s (giving 10.0 s activity overall) in which 1, 2 or 5 μM quinidine was applied immediately. Where applicable, the final 5.0 s of activity was analysed to compute the DF.

1.10. Comparison with an alternative human atria model

The human atrial cell model of Grandi *et al*. (Grandi et al., 2011), referred to in this study as the GB model, was used for comparative simulations. The GB model is largely derived from different experimental data to the CNZ model, and thus has a different AP morphology. The MC formulation of I_{Ks} was incorporated into the GB model with a conductance of 0.012 nS/pF (it should be noted that is significantly smaller than the conductance used in the CNZ model, due to inherent differences in the role of I_{Ks} between the models). This gave an APD_{90} in the WT condition of 306.0 ms, close to that of the original published model (Grandi et al., 2011). APD restitution curves were computed using the same method that was used in the CNZ model (with the membrane potential threshold adjusted to −65 mV due to the slow repolarisation tail in the GB model).

2. Supplementary Investigations

2.1. Determinants of APD restitution steepness

Supplementary simulations were conducted in order to investigate why two *KCNQ1* mutations which both cause a 'gain-of-function' in I_{Ks} produced opposite effects on the maximum slope of restitution, shown in Figure S11. These investigations revealed the following: (1) A linear increase in the maximal conductance of I_{Ks} in the WT condition, in order to match roughly the degree of APD shortening in the V307L mutation condition, increased the slope of restitution to an even greater extent than observed under V307L conditions. (2) Addition of the instantaneous voltage-independent component (ξ) in the V307L mutation condition dramatically reduced the maximum slope. (3) The maximum slope under V141M mutation conditions in which either (i) only ξ was considered or (ii) kinetics were kept the same but ξ was removed, remained much smaller than in the WT condition. These three observations suggest that increased I_{Ks} early during the AP, either due to slower deactivation under SQT2 mutation conditions or presence of a constitutively active component, reduces the maximum slope of restitution. (4) A final hypothetical V141M mutation condition without ξ and with a 2.5-fold increase in maximal conductance of I_{CaL} showed a restitution steepness close to that of the WT condition. This suggests that the effects of ξ on reducing I_{CaL} are also an important factor in determining restitution steepness, in agreement with a previous study that suggested restitution of I_{CaL} is a primary determinant of the steepness of APD restitution (Qu et al., 1999).

2.2. Comparison with an alternative human atria model

In comparative simulations using the GB model, the V141M and V307L KCNQ1 mutations produced much more modest APD shortening than in the CNZ model (due to smaller maximal I_{Ks} conductance and lesser activation of I_{Ks} during the AP; Figure S12). Nonetheless, a greater APD shortening under V141M mutation conditions than V307L mutation conditions was observed, consistent with CNZ model simulations (Supplementary Table S8). Furthermore, opposing effects on the maximal slope of APD restitution were similarly observed (decreased steepness for the V141M mutation and increased steepness for the V307L mutation). These findings are reassuring; in the CNZ model, greater APD shortening associated with the V141M mutation was responsible for the higher DF of re-entrant excitations than under V307L mutation conditions, and steepness of APD restitution in SQT2 mutation conditions mediated spiral wave dynamics. Both of these important determinants of tissue-level re-entrant wave behaviour were reproduced using the GB model – an alternative, well-established human atrial AP model (Grandi et al., 2011).

2.3. Effects of AF-induced electrical and structural remodelling on SQT2 phenotype

In the 3D anatomical human atria, the effects of quinidine on arrhythmic atrial excitation waves are shown in Manuscript Figure 7. Supplementary simulations were performed in order to investigate potential interactions between AF remodelling and *KCNQ1* mutations. When AF-induced structural/connexin remodelling (40% reduction in **D**) was included, either alone or combined with electrical remodelling, quinidine was ineffective at pharmacological conversion of arrhythmic excitation waves under both mutation conditions (Figure S13). However, quinidine still reliably decreased the DF in a dose-dependent manner.

3. Supplementary Figures

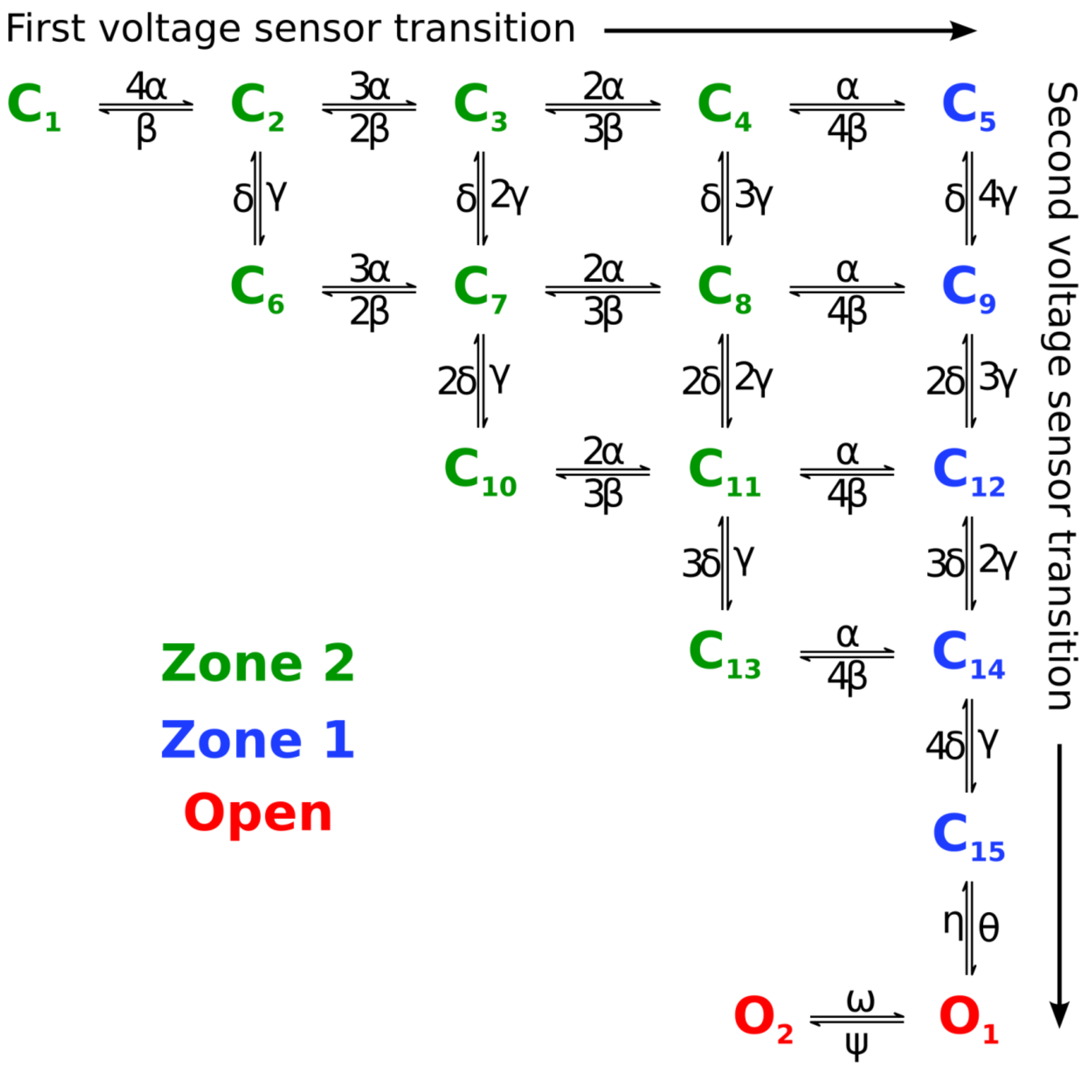

Figure S1: The Markov chain scheme for human I_{Ks}. C_1-C_{15} represent closed states, whereas O_1 and O_2 represent open states. Green closed states (zone 2) represent channels which have not completed the first voltage sensor transition for all four subunits, and blue closed states (zone 1) represent channels which have completed the four first voltage sensor transitions. The activated (open) states are shown in red.

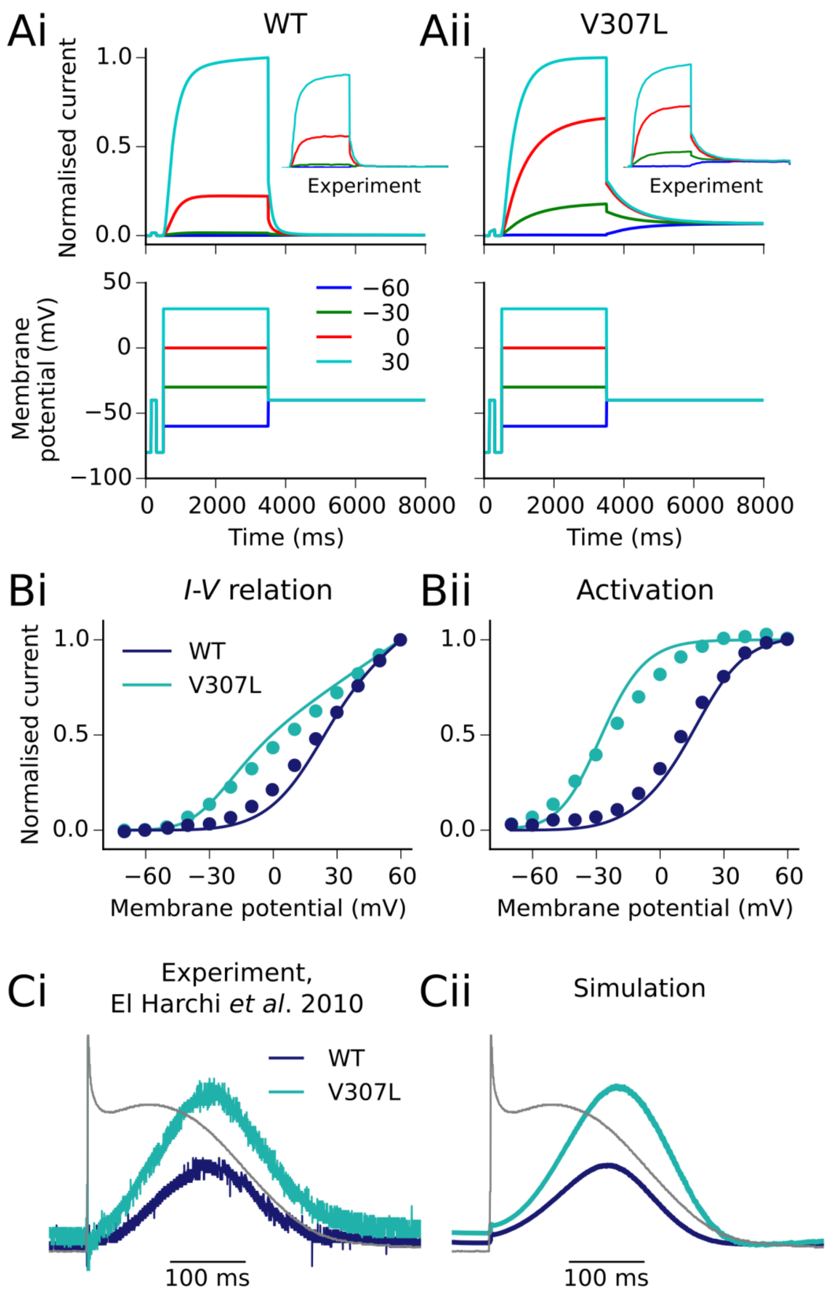

Figure S2: Simulated voltage and action potential clamp experiments under WT and V307L conditions. (A) Simulated current traces under WT (i) and V307L (ii) conditions at test potentials of −60 mV (blue), −30 mV (green), 0 mV (red), and 30 mV (cyan), following the voltage clamp protocol shown. Experimental current traces are shown inset (El Harchi et al., 2010). (B) Corresponding *I-V* relation measured at the end of step potentials (i) and voltage dependence of activation measured from peak tail currents (ii) under WT (dark blue) and V307L (cyan) conditions, where solid lines are simulation results, and points are experimental data. (C) Comparison of experimental (i) and simulated (ii) WT and V307L KCNQ1 mutant I_{Ks} current profiles from action potential (AP) clamps using the Courtemanche *et al*. (CRN) human atrial AP waveform (Courtemanche et al., 1998). Amplitudes of simulated current profiles were normalised to match those of the experimental traces to facilitate comparison. Experimental data are taken from El Harchi *et al*. at 37°C (El Harchi et al., 2010).

A WT

$$\alpha = 5.56 \cdot 10^{-5} \cdot \exp\left\{3.61 \cdot 10^{-1} \cdot \frac{Vm \cdot F}{R \cdot T}\right\}$$

$$\beta = 8.25 \cdot 10^{-6} \cdot \exp\left\{-9.23 \cdot 10^{-2} \cdot \frac{Vm \cdot F}{R \cdot T}\right\}$$

$$\gamma = 3.78 \cdot 10^{-4} \cdot \exp\left\{8.68 \cdot 10^{-1} \cdot \frac{Vm \cdot F}{R \cdot T}\right\}$$

$$\delta = 1.32 \cdot 10^{-4} \cdot \exp\left\{-3.30 \cdot 10^{-1} \cdot \frac{Vm \cdot F}{R \cdot T}\right\}$$

$$\theta = 6.10 \cdot 10^{-4}$$

$$\eta = 1.95 \cdot 10^{-3} \cdot \exp\left\{-4.81 \cdot 10^{-1} \cdot \frac{Vm \cdot F}{R \cdot T}\right\}$$

$$\psi = 6.25 \cdot 10^{-4} \cdot \exp\left\{1.27 \cdot 10^{-0} \cdot \frac{Vm \cdot F}{R \cdot T}\right\}$$

$$\omega = 3.50 \cdot 10^{-4} \cdot \exp\left\{-6.79 \cdot 10^{-1} \cdot \frac{Vm \cdot F}{R \cdot T}\right\}$$

B V307L

$$\alpha = 2.52 \cdot 10^{-5} \cdot \exp\left\{3.61 \cdot 10^{-1} \cdot \frac{Vm \cdot F}{R \cdot T}\right\}$$

$$\beta = 2.51 \cdot 10^{-5} \cdot \exp\left\{-9.23 \cdot 10^{-2} \cdot \frac{Vm \cdot F}{R \cdot T}\right\}$$

$$\gamma = 1.71 \cdot 10^{-3} \cdot \exp\left\{8.68 \cdot 10^{-1} \cdot \frac{Vm \cdot F}{R \cdot T}\right\}$$

$$\delta = 6.45 \cdot 10^{-4} \cdot \exp\left\{-3.30 \cdot 10^{-1} \cdot \frac{Vm \cdot F}{R \cdot T}\right\}$$

$$\theta = 3.40 \cdot 10^{-3}$$

$$\eta = 4.75 \cdot 10^{-4} \cdot \exp\left\{-4.81 \cdot 10^{-1} \cdot \frac{Vm \cdot F}{R \cdot T}\right\}$$

$$\psi = 1.93 \cdot 10^{-3} \cdot \exp\left\{1.27 \cdot 10^{0} \cdot \frac{Vm \cdot F}{R \cdot T}\right\}$$

$$\omega = 4.52 \cdot 10^{-4} \cdot \exp\left\{-6.79 \cdot 10^{-1} \cdot \frac{Vm \cdot F}{R \cdot T}\right\}$$

C V141M

$$\alpha = 2.03 \cdot 10^{-4} \cdot \exp\left\{2.60 \cdot 10^{-1} \cdot \frac{V_m \cdot F}{R \cdot T}\right\}$$

$$\beta = 2.70 \cdot 10^{-5} \cdot \exp\left\{-1.26 \cdot 10^{-1} \cdot \frac{V_m \cdot F}{R \cdot T}\right\}$$

$$\gamma = 1.25 \cdot 10^{-2} \cdot \exp\left\{5.68 \cdot 10^{-1} \cdot \frac{V_m \cdot F}{R \cdot T}\right\}$$

$$\delta = 8.59 \cdot 10^{-4} \cdot \exp\left\{-3.66 \cdot 10^{-1} \cdot \frac{V_m \cdot F}{R \cdot T}\right\}$$

$$\theta = 1.03 \cdot 10^{-2}$$

$$\eta = 2.92 \cdot 10^{-3} \cdot \exp\left\{-4.59 \cdot 10^{-1} \cdot \frac{V_m \cdot F}{R \cdot T}\right\}$$

$$\psi = 4.89 \cdot 10^{-3} \cdot \exp\left\{2.14 \cdot 10^{0} \cdot \frac{V_m \cdot F}{R \cdot T}\right\}$$

$$\omega = 5.46 \cdot 10^{-6} \cdot \exp\left\{-9.79 \cdot 10^{-1} \cdot \frac{V_m \cdot F}{R \cdot T}\right\}$$

$$\xi = 2.29 \cdot 10^{-2}$$

Figure S3: Markov chain parameters for WT and SQT2 mutant I_{Ks}. Transition rates in (A) WT, (B) V307L, and (C) V141M conditions. Parameters are defined as follows: V_m is the membrane potential; R is the molar gas constant; T is the temperature; F is the Faraday constant. All parameters correspond to a temperature of 37°C. All transition rates have units of ms^{-1}.

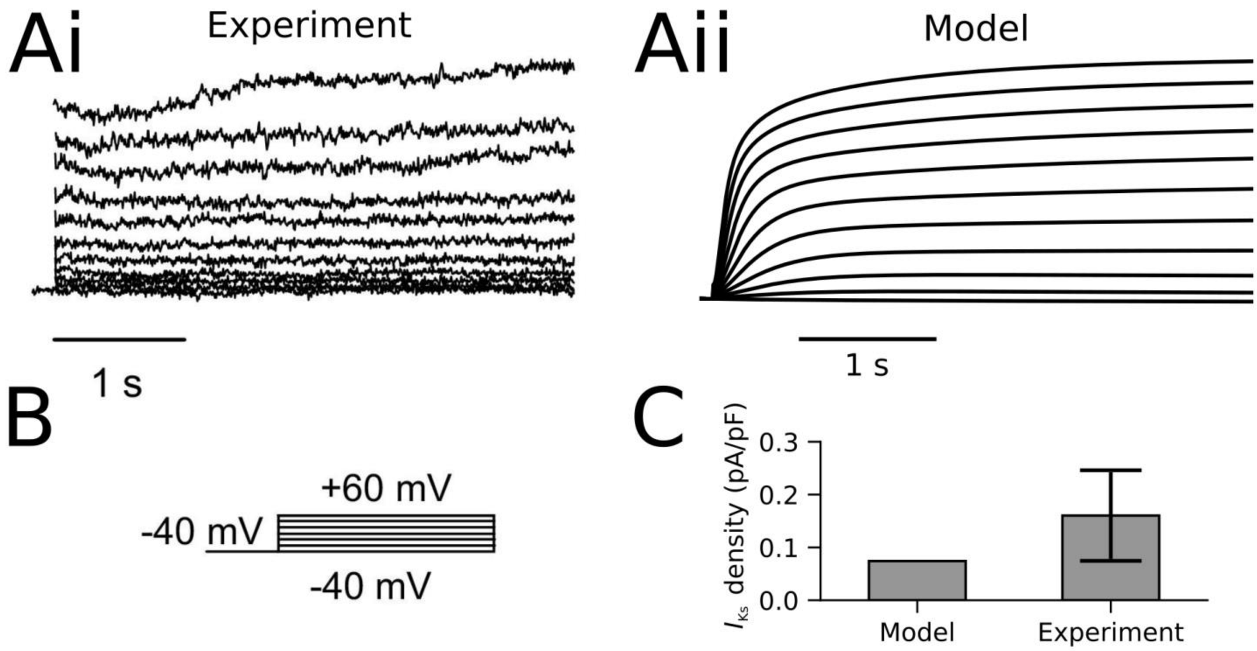

Figure S4: Comparison of human atrial I_{Ks}: experiment vs. model. (Ai) Experimental and (Aii) simulated measurements of human atrial I_{Ks}, using the protocol shown in (B). (C) A comparison of I_{Ks} current density between model and experiment – data taken from undiseased human atrial myocytes (Caballero et al., 2010).

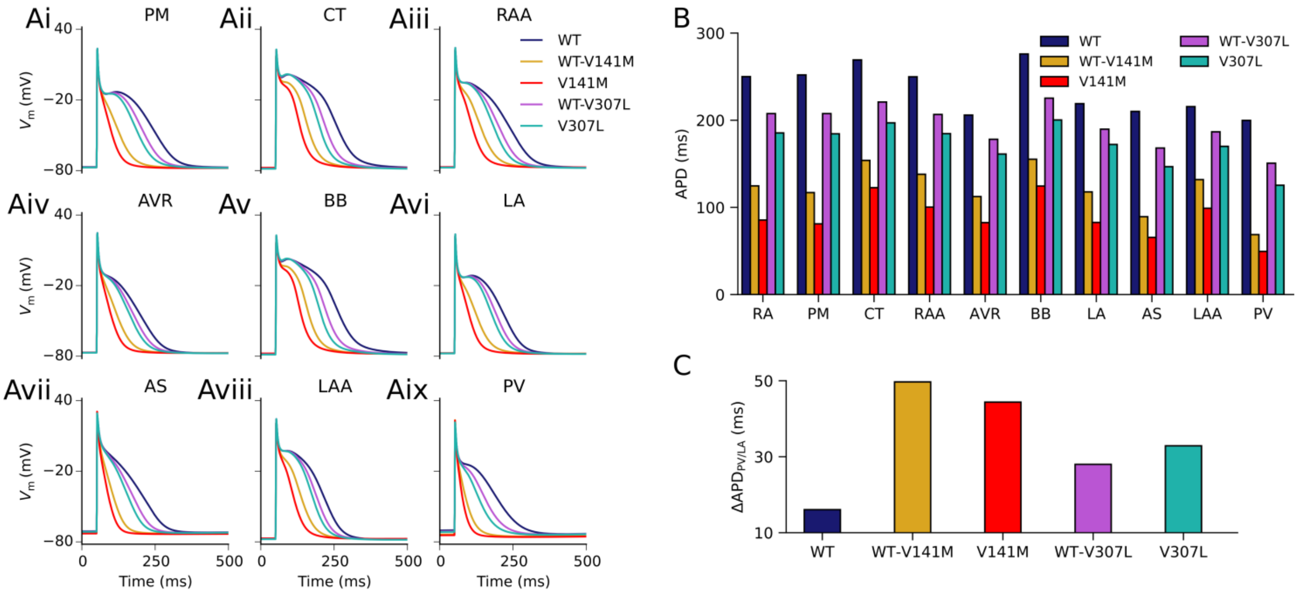

Figure S5: Response of atrial regional cell models to *KCNQ1* mutants. Action potential profiles in WT, WT-V141M, V141M, WT-V307L, and V307L conditions at 1 Hz in the pectinate muscles (PM; Ai), crista terminalis (CT; Aii), right atrial appendage (RAA; Aiii), atrio-ventricular ring (AVR; Aiv), Bachmann's bundle (BB; Av), left atrium (LA; Avi), atrial septum (AS; Avii), left atrial appendage (LAA; Aviii), and pulmonary vein (PV; Aix) regions of the CNZ human atrial cell model. (B) A barchart summary of the action potential duration (APD) in different regional cell models. (C) Dispersion of APD between PV/LA cells under WT and SQT2 mutation conditions.

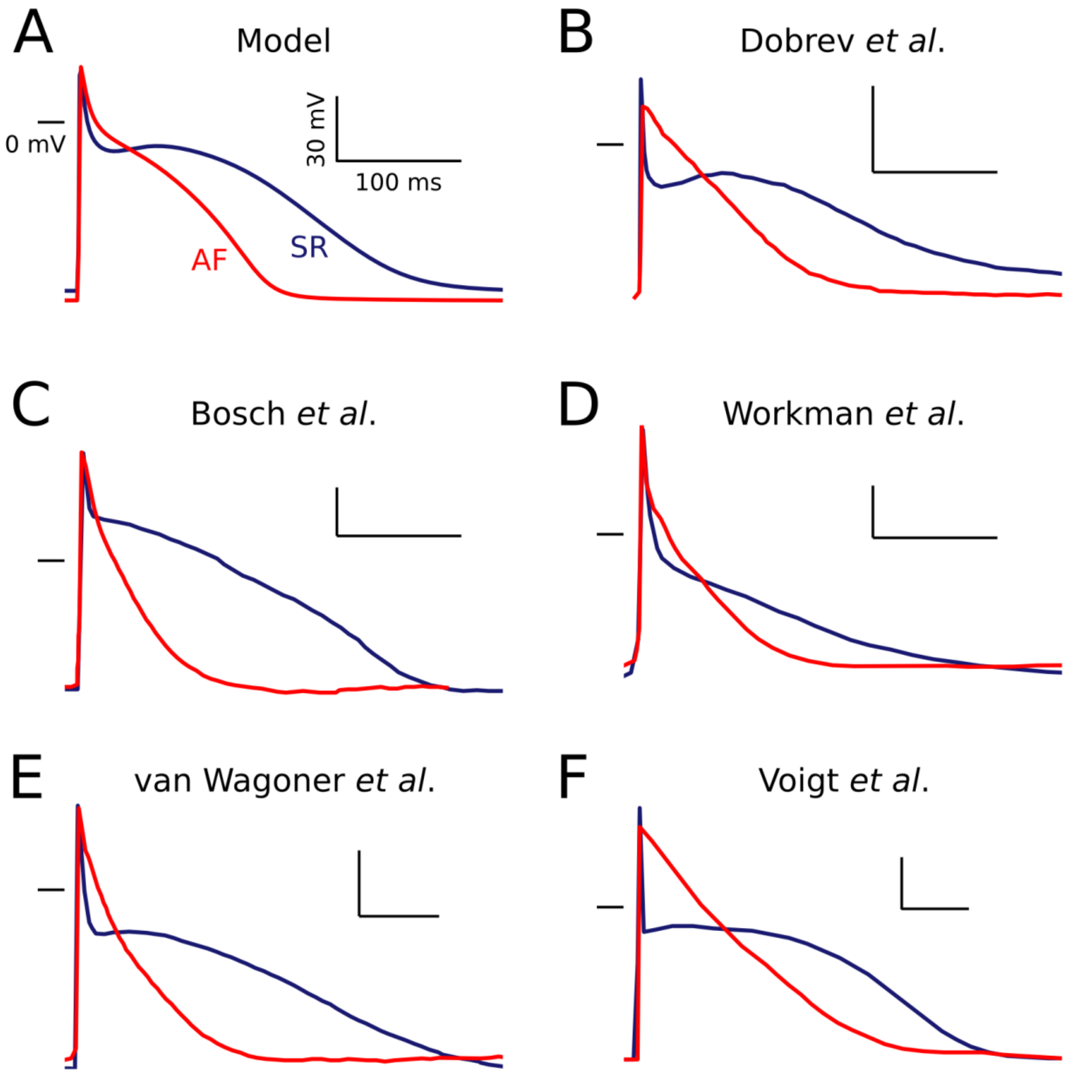

Figure S6: Comparison of AF model with experimental data. The CNZ model AP under normal sinus rhythm (SR) and atrial fibrillation (AF) remodelling conditions at 1 Hz (A) compared with experimentally-measured human atrial APs from (B) Dobrev *et al*. (Dobrev and Ravens, 2003), (C) Bosch *et al*. (Bosch et al., 1999), (D) Workman *et al*. (Workman et al., 2001), (E) van Wagoner *et al*. (Van Wagoner and Nerbonne, 2000), and (F) Voigt *et al*. (Voigt et al., 2012). In all panels, a marker to the left of the APs represents a membrane potential of 0 mV, and the scale inset corresponds to 100 ms and 30 mV along the *x* and *y* axes, respectively.

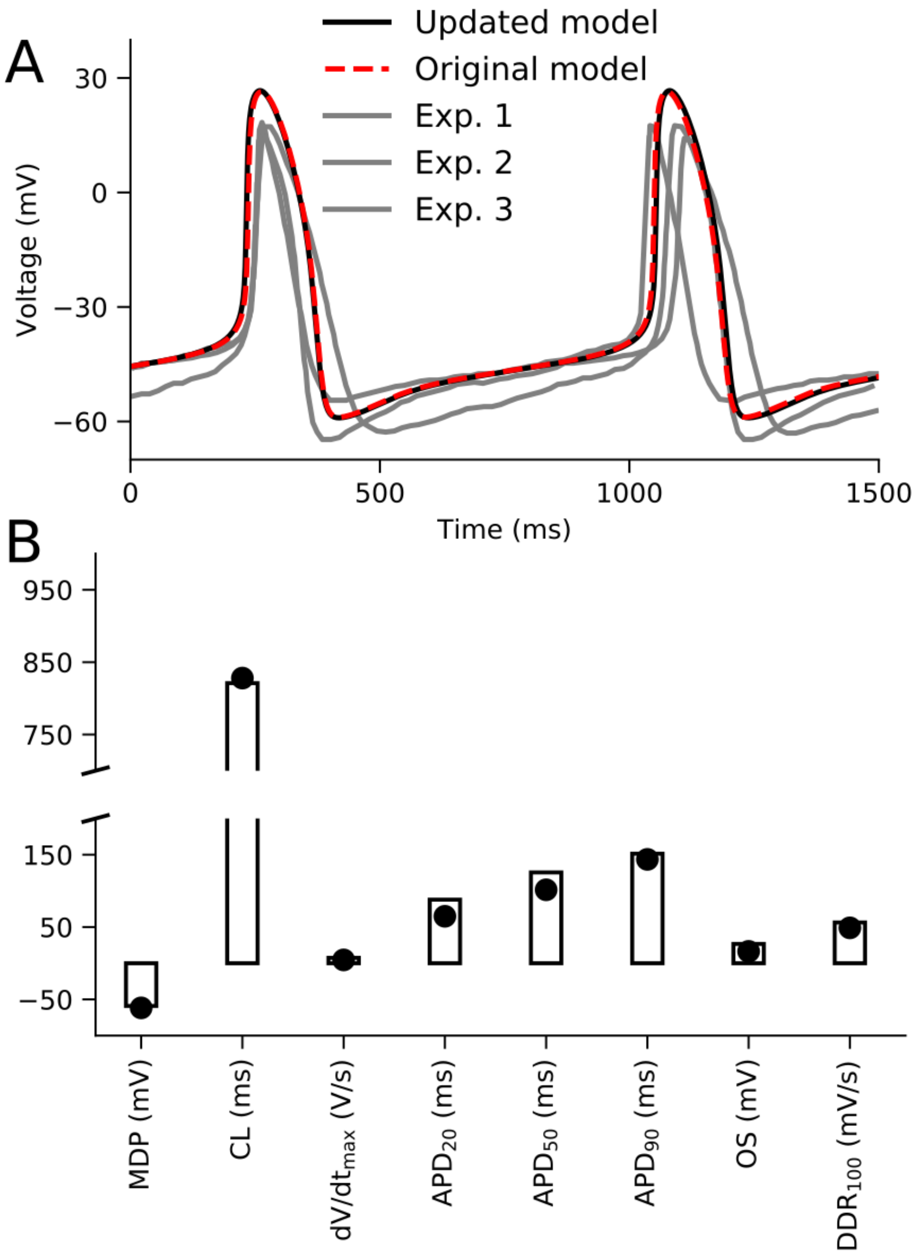

Figure S7: Comparison of the modified FS human sinoatrial node model to experimental data. (A) Simulated action potential (AP) of a single human sinoatrial node (SAN) myocyte using updated model in this study (black, solid line) compared with original model (red, dashed line), and three experimentally-recorded APs (grey, solid lines) from isolated human SAN cells (Verkerk et al., 2007). (B) A comparison of AP metrics between model (points) and experiment (bars); namely, the maximum diastolic potential (MDP), cycle length (CL), action potential duration at 20%, 50%, and 90% (APD_{20}, APD_{50}, and APD_{90}, respectively), overshoot (OS), and diastolic depolarisation rate at 100 ms (DDR_{100}). All experimental data are taken from (Verkerk et al., 2007).

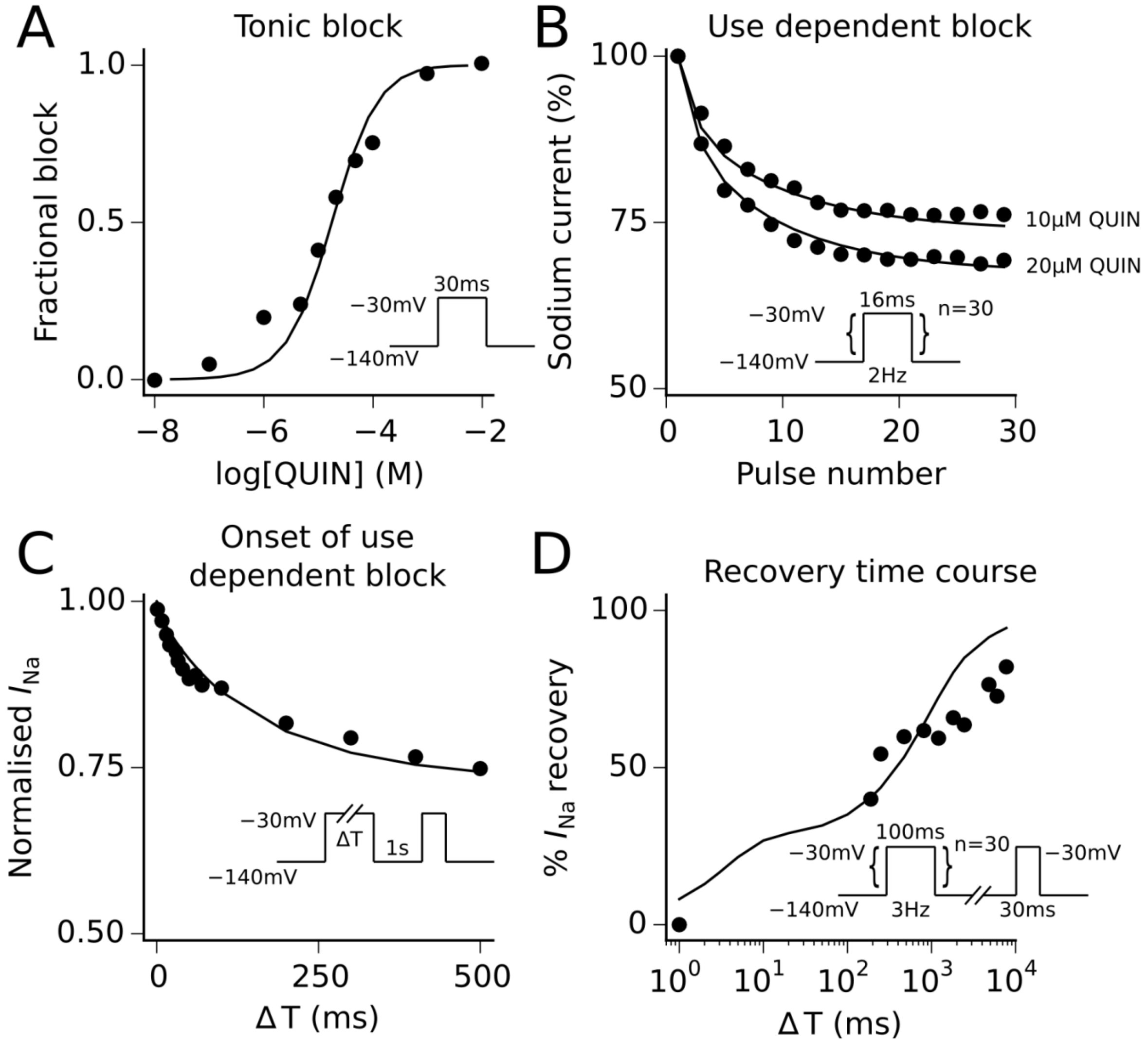

Figure S8: Modelling quinidine interactions with sodium channels. (A) Simulated (solid lines) and experimental (points) tonic block of I_{Na} by quinidine (QUIN) using the pulse protocol shown inset. (B) Use-dependent block of I_{Na} by 10 μM and 20 μM QUIN, measured using the pulse protocol shown inset (n = 30 pulses). (C) Onset of use dependent block measured using pulse protocol shown inset with depolarising pulses of varying duration, ΔT. (D) Recovery time course of sodium channels measured using repeated depolarising pulses (n = 30) followed by a repolarising pulse of varying duration, ΔT. Experimental data were recorded in mammalian cardiac myocytes (Koumi et al., 1992), with a reported tonic block IC_{50} value of 17 μM.

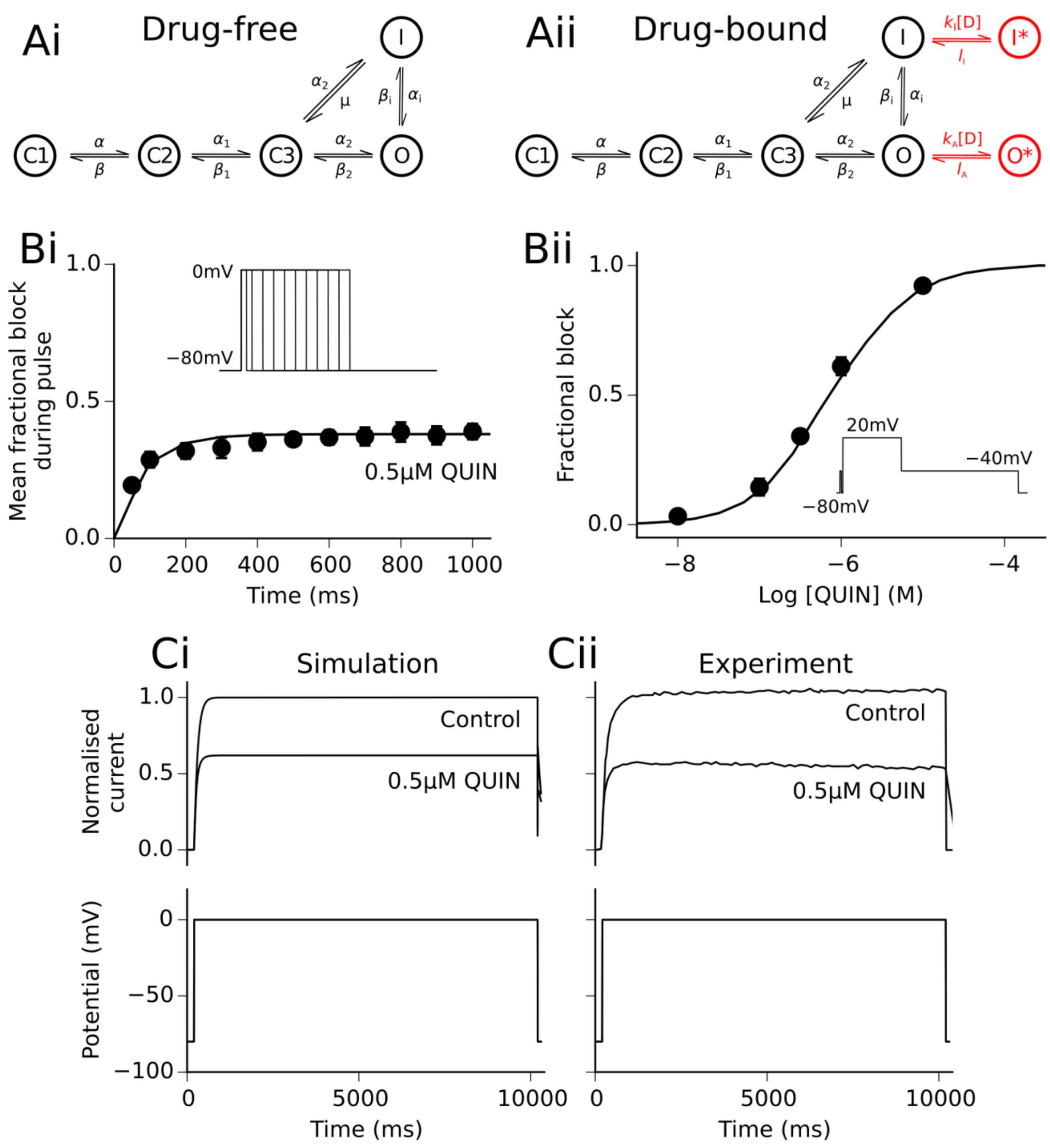

Figure S9: Modelling quinidine interactions with I_{Kr}/hERG channels. (Ai) Drug-free and (Aii) drug-bound (additional states shown in red) Markov chain models of I_{Kr}/hERG. Simulated (solid line) and experimental (points) mean fractional block by 0.5 μM quinidine (QUIN) of I_{hERG} during pulse protocol (shown inset) (Bi), and dose-response curve (Bii), where the IC_{50} value is 620 nM. (Ci) Simulated and (Cii) experimental current traces in response to a 10,000 ms voltage step to 0 mV from a holding potential of −80 mV under control conditions and with application of 0.5 μM QUIN. Experimental data at 37°C are taken from (McPate et al., 2008; Paul et al., 2002).

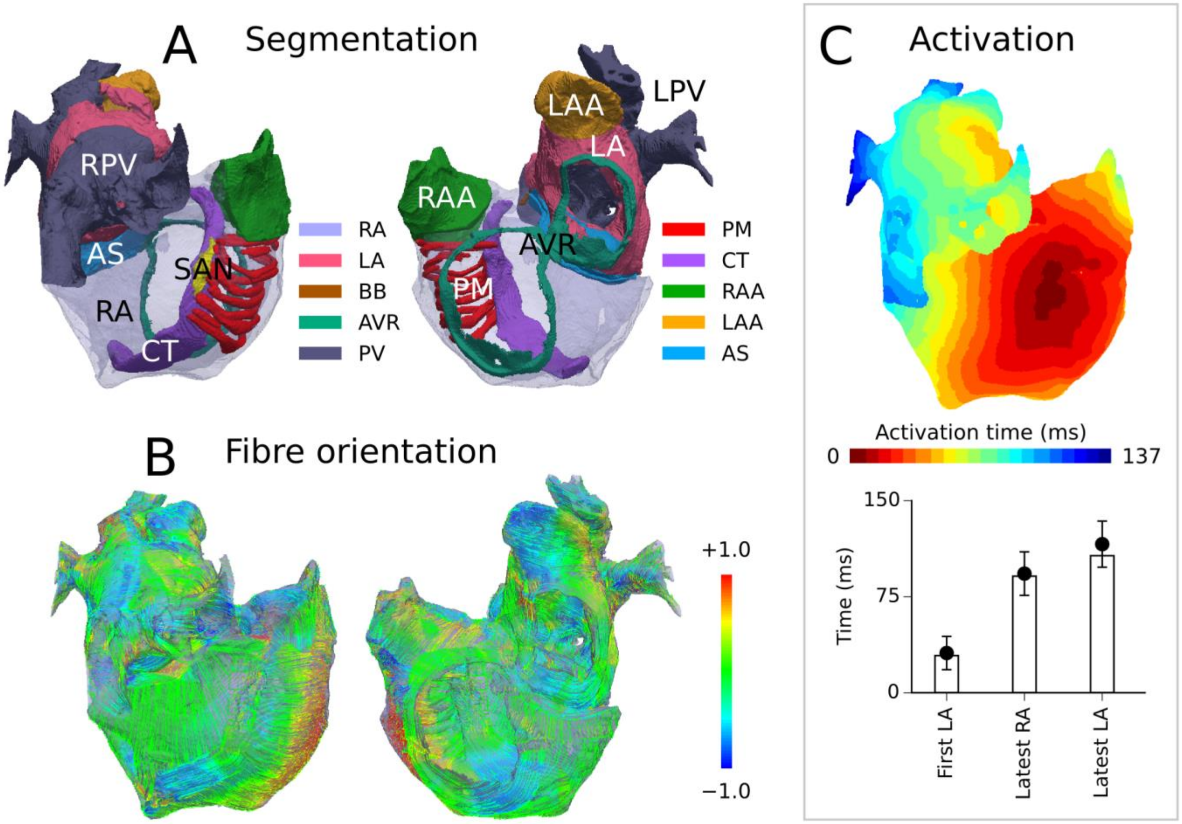

Figure S10: 3D anatomical human atria model. The 3D anatomical human atria geometry shown from a right atrial posterior wall view and looking into the cavities (A), with the segmented regions of the atrial septum (AS), atrio-ventricular ring (AVR), crista terminalis (CT), left atrium (LA), left atrial appendage (LAA), left pulmonary veins (LPV), pectinate muscles (PM), right atrium (RA), right atrial appendage (RAA), right pulmonary veins (RPV), and sinoatrial node (SAN) shown for reference – Bachmann's bundle (BB) not shown. (B) Traced fibre orientations, where the colour map corresponds to the *z* component (vertical direction) of the longitudinal vector of the fibres. (C) The activation sequence following stimulation of the SAN region, with model activation times (bars) compared with the experimental data (points) of Lemery *et al*. (Lemery et al., 2007).

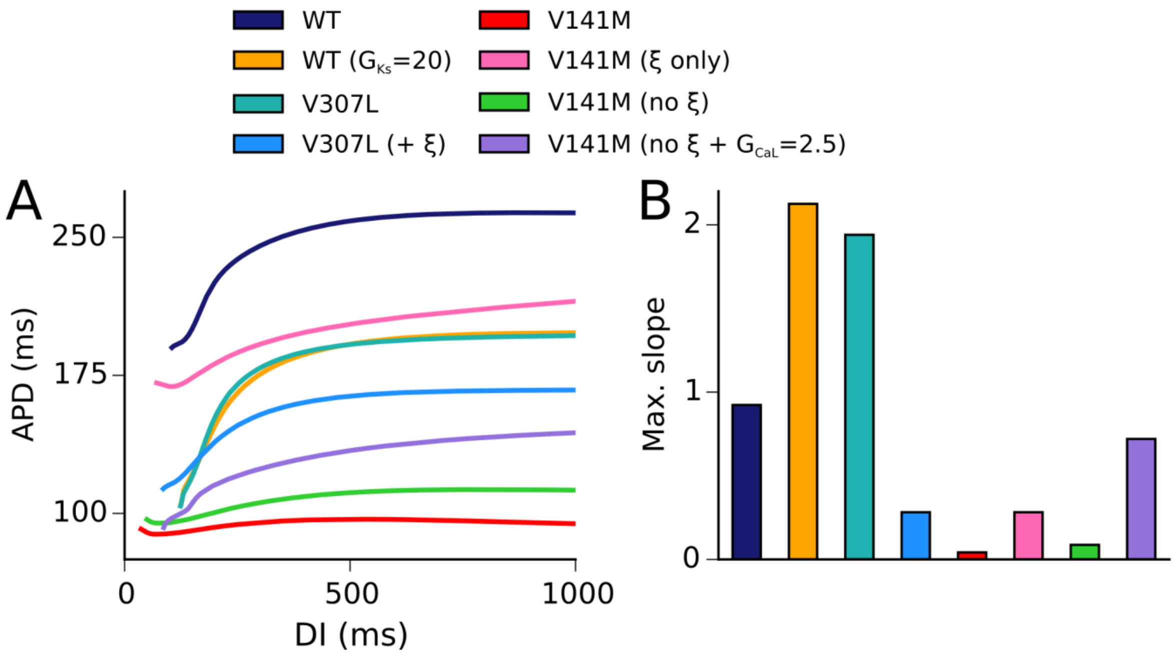

Figure S11: Determinants of the steepness of APD restitution. (A) Restitution of the APD measured at −70 mV using an S1-S2 protocol under the following conditions: WT (dark blue); WT with a 20-fold increase in I_{Ks} maximal conductance (G_{Ks}) (orange); V307L (teal); V307L plus an instantaneous voltage-independent component (ξ) (light blue); V141M (red); V141M with WT kinetics plus ξ (pink); V141M without ξ (green); and V141M without ξ and with a 2.5-fold increase in I_{CaL} maximal conductance (G_{CaL}) (purple). (B) A bar chart showing the corresponding maximal slope of restitution.

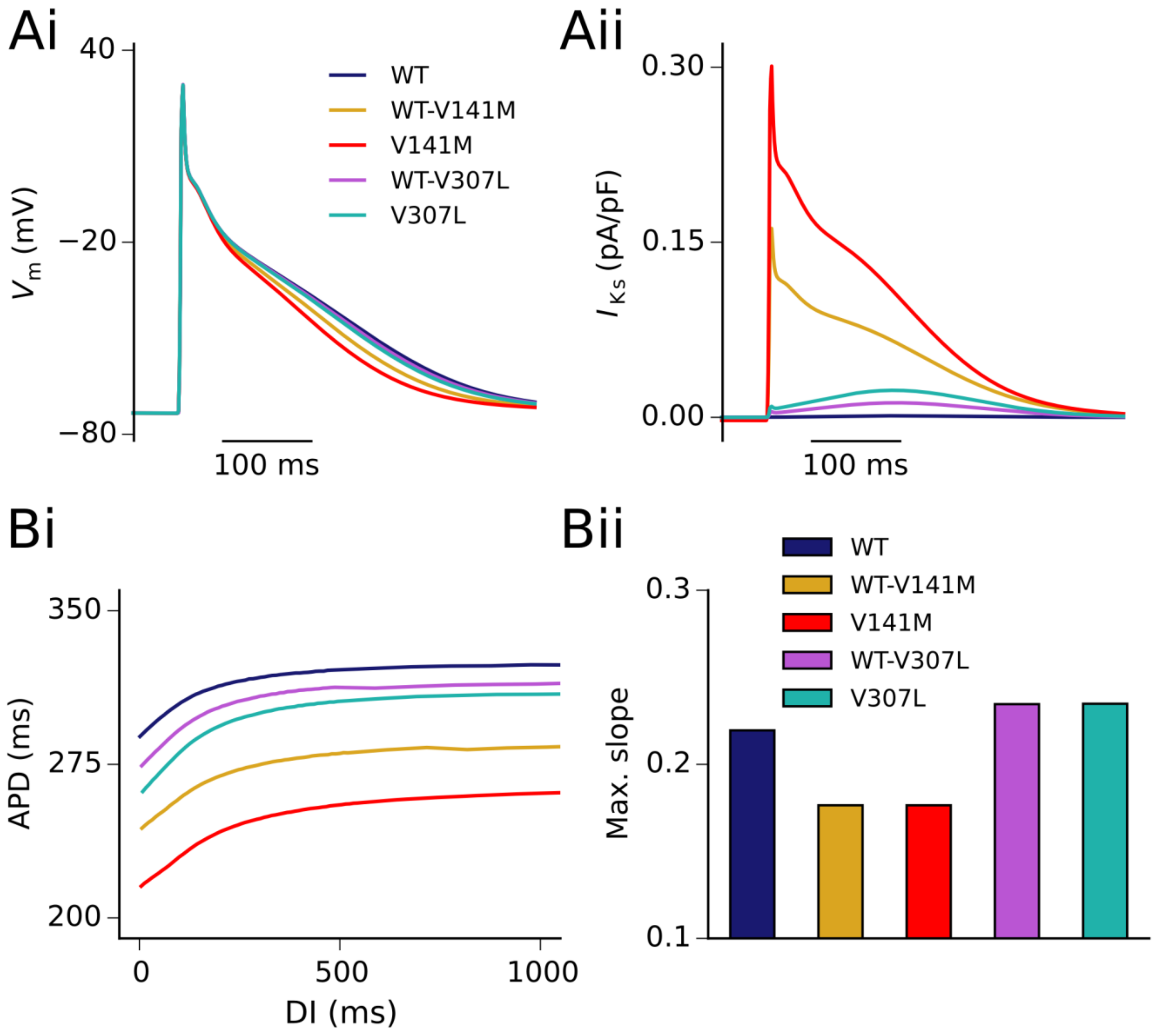

Figure S12: AP and I_{Ks} profile in WT and SQT2 mutation conditions using the GB model. Action potential waveforms in WT (dark blue), WT-V141M (gold) V141M (red), WT-V307L (magenta), and V307L (teal) conditions at a pacing frequency of 1 Hz (Ai), with corresponding current trace for I_{Ks} (Aii). Restitution of the APD measured at −65 mV using an S1-S2 protocol (Bi), and a bar chart showing maximal slope of restitution (Bii).

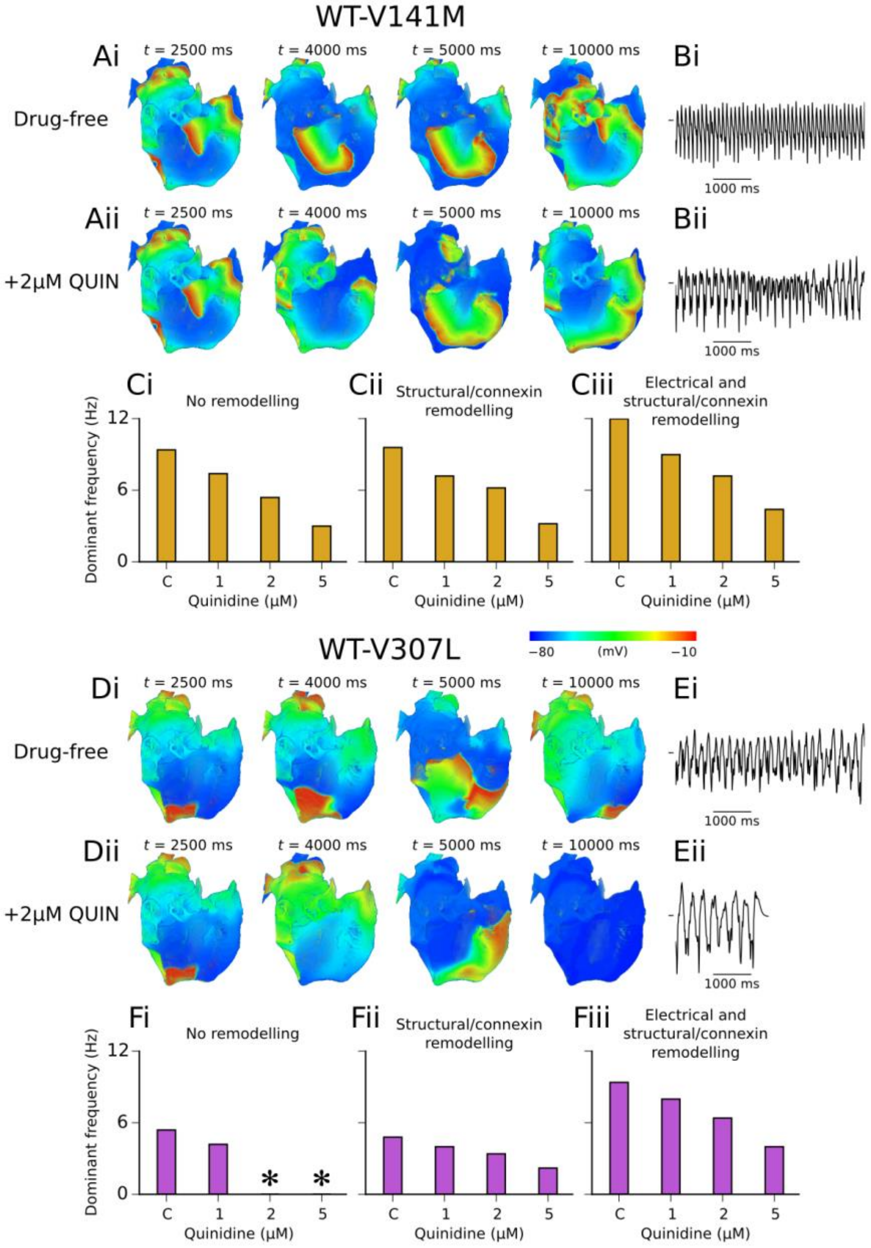

Figure S13: Effects of quinidine on re-entrant waves in SQT2 mutation conditions. (A) Snapshots of re-entrant scroll at various time points after initiation of a scroll wave in the 3D virtual human atria under drug-free WT-V141M (i), and WT-V141M + 2μM quinidine (QUIN) (ii) conditions (with no remodelling), with (B) corresponding pseudo ECG (pECG) recorded during the final 5.0 s. (C) Dominant frequencies in control (c) WT-V141M conditions and upon application of various concentrations of quinidine with no remodelling (i), structural/connexin remodelling (ii), and combined electrical and structural/connexin remodelling (iii). (D) Snapshots of re-entrant scroll waves following re-entry initiation under drug-free WT-V307L (i), and WT-V307L + 2μM QUIN (ii) conditions, with (E) corresponding pECGs recorded during the final 5.0 s. (E) Dominant frequencies in control (c) WT-V307L conditions and upon application of quinidine with no remodelling (i), structural/connexin remodelling (ii), and combined electrical and structural/connexin remodelling (iii). In drug simulations QUIN was applied at t = 2500 ms. * denotes that re-entrant scroll waves self-terminated.

4. Supplementary Tables

Table S1. Comparison of model APD with experimental measurements.

Study	APD_{90} (ms) at 1 Hz
This study	250
Bosch *et al*. (Bosch et al., 1999)	255±45
Dobrev *et al*. (Dobrev and Ravens, 2003)	203±10
Katoh *et al*. (Katoh et al., 2005)	255±39
Kim *et al*. (Kim et al., 2002)	258±25
Pau *et al*. (Pau et al., 2007)	239±11
Redpath *et al*. (Calum J Redpath, 2006)	210±17

A summary of APD_{90} values from a range of experimental measurement from human atrial myocytes, as well as the model used in this study.

Table S2. Ionic differences in the CNZ family of regional cell models.

	G_{CaL}	G_{to}	G_{Kur}	G_{Na}	G_{Kr}	G_{Ks}	G_{K1}	Source
**CT**	2.0	1.0	1.0	1.0	1.0	1.0	1.0	(Feng et al., 1998)
**BB**	2.2	1.0	1.0	1.0	1.0	1.0	1.0	(Burashnikov et al., 2004; Feng et al., 1998)
**PM**	0.94	1.0	1.0	1.0	1.0	1.0	1.0	(Feng et al., 1998)
**AVR**	0.67	0.6	1.0	1.0	1.63	1.0	1.0	(Feng et al., 1998)
**RAA**	1.0	0.68	1.0	1.0	1.0	1.0	1.0	(Feng et al., 1998; Gong et al., 2008)
**AS**	0.4	0.212	0.667	1.3	1.0	1.0	$V_{1/2}$ −6	(Gong et al., 2008)
**LA**	1.0	1.0	1.0	1.0	1.6	1.0	1.0	(Ehrlich et al., 2003; Feng et al., 1998; Li et al., 2001)
**LAA**	1.0	0.68	0.8	1.0	1.6	1.0	1.0	(Caballero et al., 2010; Feng et al., 1998; Li et al., 2001)
**PV**	0.7	0.75	1.0	1.0	2.4	2.0	0.67	(Cha et al., 2005; Datino et al., 2010; Ehrlich et al., 2003)

A summary of conductance scaling factors, G_X, for maximal conductance of ionic current I_X relative to the baseline (RA) cell model and corresponding experimental data sources. Abbreviations are as follows: CT = crista terminalis, BB = Bachmann's bundle, PM = pectinate muscles, AVR = atrio-ventricular ring, RAA = right atrial appendage, AS = atrial septum, LA = left atrium, LAA = left atrial appendage, PV = pulmonary veins.

Table S3: Model parameters describing AF remodelling conditions.

Process	AF model	Experimental observation
I_{CaL}	−65%	−65% (Workman et al., 2001); −70% (Bosch et al., 1999); −63% (Wagoner et al., 1999)
I_{to}	−65%	−65% (Workman et al., 2001); −70% (Bosch et al., 1999); −66% (Wagoner et al., 1999)
I_{K1}	+110%	+75% (Workman et al., 2001); +100% (Bosch et al., 1999); +106% (Wagoner et al., 1999); +137% (Dobrev et al., 2001)
I_{Kur}	−50%	−49% (Wagoner et al., 1997); −45% LA (Caballero et al., 2010); −55% RA (Caballero et al., 2010); −60% (Christ et al., 2004)
I_{Ks}	+100%	+100% LA (Caballero et al., 2010); >+100% RA (Caballero et al., 2010)
I_{NaCa}	+40%	+43±14% (Neef et al., 2010); +67% (Schotten et al., 2002)
$K_{SR,leak}$	+50%	Increased (Voigt et al., 2012)
**RYR release**	+200%	Open probability increased (Voigt et al., 2012)

A summary of model parameters describing AF remodelling conditions for different processes, and corresponding experimental observations. The AF model implemented changes relative to the baseline CNZ model. LA and RA are left and right atrium, respectively.

Table S4: Secondary pharmacological effects of quinidine on ion channels.

	IC_{50} (μM)	Source
I_{CaL}	14.9	(Zhang and Hancox, 2002)
I_{to}	21.8	(Nenov et al., 1998)
I_{Ks}	44.0	(Kang et al., 2001)
I_{K1}	42.6	(Nenov et al., 1998)
I_{NaL}	12.0	(Wu et al., 2008)
I_{Kur}	6.6	(Nenov et al., 1998)

A summary of half maximal inhibitory concentration values (IC_{50}) extracted from the literature. Block of I_{NaL} (highlighted in red) by quinidine was not included in simulations, as late sodium current is not included in the CNZ model.

Table S5: Rate transitions for the guarded receptor model of quinidine binding to I_{Na}.

	**Quinidine**
k_A (μM^{-1}s^{-1})	1.87×10^{0}
l_A (s^{-1})	1.73×10^{-7}
k_I (μM^{-1}s^{-1})	8.90×10^{-1}
l_I (s^{-1})	1.12×10^{-6}
k_R (μM^{-1}s^{-1})	2.58×10^{-1}
l_R (s^{-1})	4.60×10^{-6}
**Open state affinity (μM)**	9.23×10^{-2}
**Inactivated state affinity (μM)**	1.26×10^{0}
**Resting state affinity (μM)**	1.78×10^{1}

Binding (*k*) and unbinding (*l*) rates to drug-bound activated (A), inactivated (I), and resting (R) states corresponding to the guarded receptor model equations. Drug affinities for states of type X were determined by computing l_X/k_X.

Table S6: Rate transitions to drug-bound states of the Markov chain scheme for I_{Kr}.

	**Quinidine**
k_A (μM^{-1}s^{-1})	1.44×10^{3}
l_A (s^{-1})	4.37×10^{-3}
k_I (μM^{-1}s^{-1})	1.91×10^{2}
l_I (s^{-1})	1.29×10^{-5}
**Open state affinity (μM)**	3.02×10^{0}
**Inactivated state affinity (μM)**	6.75×10^{-1}

Binding (*k*) and unbinding (*l*) rates to drug-bound activated (A) and inactivated (I) states corresponding to the drug-bound Markov chain scheme presented in Figure S8. Drug affinities for states of type X were determined by computing l_X/k_X.

Table S7: A summary of APD/ERP prolongation in SQT2 by quinidine.

	APD, 1 Hz (ms)	APD, 2 Hz (ms)	ERP, 1 Hz (ms)	ERP, 2 Hz (ms)
**WT**	250.0	219.8	274	250
**WT-V141M (+AF)**	124.7 (107.4)	88.5 (82.4)	172 (135)	129 (109)
**+ 1µM QUIN**	142.2 (116.8)	101.2 (89.9)	191 (146)	148 (118)
**+ 2µM QUIN**	152.4 (123.6)	110.6 (95.7)	207 (154)	164 (126)
**+ 5µM QUIN**	167.6 (137.3)	127.3 (108.1)	274 (181)	222 (157)
**WT-V307L (+AF)**	207.6 (152.2)	182.4 (133.4)	233 (174)	212 (158)
**+ 1µM QUIN**	229.6 (174.4)	207.5 (152.4)	264 (195)	244 (177)
**+ 2µM QUIN**	243.0 (189.6)	222.5 (166.5)	290 (211)	273 (192)
**+ 5µM QUIN**	268.8 (216.6)	251.4 (193.6)	390 (262)	392 (246)

Action potential duration (APD) and effective refractory period (ERP) prolongation by various concentrations of quinidine (QUIN) both without (black) and with (red, in brackets) the effects of AF-induced electrical remodelling.

Table S8: Atrial cell AP properties in WT and SQT2 mutant conditions in the GB model.

	WT	WT-V141M	V141M	WT-V307L	V307L
**APD_{90} (ms)**	306.0	267.9	244.2	298.1	291.9
**APD_{50} (ms)**	69.0	62.4	57.8	67.9	67.6
**APA (mV)**	102.5	102.5	102.6	102.7	102.7
**MUV (V/s)**	108.0	109.6	111.3	106.0	107.6

A summary of single human atrial cell AP characteristics under WT and SQT2 mutation conditions at a pacing frequency of 1 Hz in the GB model. Abbreviations are: action potential duration at 50% and 90% repolarisation (APD_{50} and APD_{90}, respectively), action potential amplitude (APA), and maximum upstroke velocity (MUV).

5. Supplementary Videos

Video S1: Initiation and conduction of spiral waves in a 2D idealised geometry in the WT condition. Re-entry was initiated using an S1-S2 protocol: following propagation of a planar wave elicited with four conditioning S1 stimuli at a BCL of 400 ms, an S2 stimulus was applied in the lower left quadrant of the patch. The initiated spiral wave meanders out of the tissue boundaries after ~3.7 s.

Video S2: Initiation and conduction of spiral waves in a 2D idealised geometry in the WT-V141M condition. Re-entry was initiated using an S1-S2 protocol: following propagation of a planar wave elicited with four conditioning S1 stimuli at a BCL of 400 ms, an S2 stimulus was applied in the lower left quadrant of the patch. The initiated spiral wave persists as a stationary, stable wave for the duration of the simulation.

Video S3: Initiation and conduction of spiral waves in a 2D idealised geometry in the V141M condition. Re-entry was initiated using an S1-S2 protocol: following propagation of a planar wave elicited with four conditioning S1 stimuli at a BCL of 400 ms, an S2 stimulus was applied in the lower left quadrant of the patch. The initiated spiral wave persists as a stationary, stable wave for the duration of the simulation.

Video S4: Initiation and conduction of spiral waves in a 2D idealised geometry in the WT-V307L condition. Re-entry was initiated using an S1-S2 protocol: following propagation of a planar wave elicited with four conditioning S1 stimuli at a BCL of 400 ms, an S2 stimulus was applied in the lower left quadrant of the patch. The initiated spiral wave persists as a meandering but stable wave for the duration of the simulation.

Video S5: Initiation and conduction of spiral waves in a 2D idealised geometry in the V307L condition. Re-entry was initiated using an S1-S2 protocol: following propagation of a planar wave elicited with four conditioning S1 stimuli at a BCL of 400 ms, an S2 stimulus was applied in the lower left quadrant of the patch. The initiated spiral wave spontaneously degenerates into multiple waves, persisting for the duration of the simulation.

Video S6: Re-entrant scroll waves in the WT condition initiated in the 3D anatomical human atria model shown from two views–looking at the RA posterior wall (left) and into the cavities (right). A single scroll wave completes two circuits in the RA before self-terminating after ~0.6 s.

Video S7: Re-entrant scroll waves in the WT-V141M condition initiated in the 3D anatomical human atria model shown from two views–looking at the RA posterior wall (left) and into the cavities (right). Re-entry is driven by a single stationary scroll wave in the RA which follows a circuit along the junction of the crista terminalis and pectinate muscles, persisting for the duration of the simulation.

Video S8: Re-entrant scroll waves in the V141M condition initiated in the 3D anatomical human atria model shown from two views–looking at the RA posterior wall (left) and into the cavities (right). Re-entry is driven by a single stationary scroll wave in the right atrial appendage, persisting for the duration of the simulation.

Video S9: Re-entrant scroll waves in the WT-V307L condition initiated in the 3D anatomical human atria model shown from two views–looking at the RA posterior wall (left) and into the cavities (right). Scroll waves meander significantly throughout the atria, before settling into a persistent, anatomical re-entry around the opening of the inferior vena cava which lasts for the remainder of the simulation.

Video S10: Re-entrant scroll waves in the V307L condition initiated in the 3D anatomical human atria model shown from two views–looking at the RA posterior wall (left) and into the cavities (right). The initiated scroll wave moves unpredictably at first, then occasionally breaks and forms multiple wavelets which meander and collide. Re-entry ultimately settles into a persistent, anatomical re-entry around the opening of the inferior vena cava which lasts for the remainder of the simulation.

Video S11: Re-entrant scroll waves in the WT-V307L condition under application of 2 μM quinidine in the 3D anatomical human atria model shown from two views–looking at the RA posterior wall (left) and into the cavities (right). Following application of quinidine, the wavelength of re-entrant excitations is increased, which leads to termination of re-entry after ~7.5 s.

References

Adeniran, I., Whittaker, D. G., Harchi, A. E., Hancox, J. C., and Zhang, H. (2017). In silico investigation of a KCNQ1 mutation associated with short QT syndrome. *Sci. Rep.* 7, 8469. doi:10.1038/s41598-017-08367-2.

Biktashev, V. N., and Holden, A. V. (1998). Reentrant waves and their elimination in a model of mammalian ventricular tissue. *Chaos Interdiscip. J. Nonlinear Sci.* 8, 48–56. doi:10.1063/1.166307.

Bosch, R. F., Zeng, X., Grammer, J. B., Popovic, K., Mewis, C., and Kühlkamp, V. (1999). Ionic mechanisms of electrical remodeling in human atrial fibrillation. *Cardiovasc. Res.* 44, 121–131. doi:10.1016/S0008-6363(99)00178-9.

Bray, M.-A., and Wikswo, J. P. (2002). Use of topological charge to determine filament location and dynamics in a numerical model of scroll wave activity. *IEEE Trans. Biomed. Eng.* 49, 1086–1093. doi:10.1109/TBME.2002.803516.

Brennan, T., Fink, M., and Rodriguez, B. (2009). Multiscale modelling of drug-induced effects on cardiac electrophysiological activity. *Eur. J. Pharm. Sci.* 36, 62–77. doi:10.1016/j.ejps.2008.09.013.

Burashnikov, A., Mannava, S., and Antzelevitch, C. (2004). Transmembrane action potential heterogeneity in the canine isolated arterially perfused right atrium: effect of IKr and IKur/Ito block. *Am. J. Physiol. - Heart Circ. Physiol.* 286, H2393–H2400. doi:10.1152/ajpheart.01242.2003.

Caballero, R., de la Fuente, M. G., Gómez, R., Barana, A., Amorós, I., Dolz-Gaitón, P., et al. (2010). In Humans, Chronic Atrial Fibrillation Decreases the Transient Outward Current and Ultrarapid Component of the Delayed Rectifier Current Differentially on Each Atria and Increases the Slow Component of the Delayed Rectifier Current in Both. *J. Am. Coll. Cardiol.* 55, 2346–2354. doi:10.1016/j.jacc.2010.02.028.

Calum J Redpath, A. C. R. (2006). Anti-adrenergic effects of endothelin on human atrial action potentials are potentially anti-arrhythmic. *J. Mol. Cell. Cardiol.* 40, 717–24. doi:10.1016/j.yjmcc.2006.01.012.

Cha, T.-J., Ehrlich, J. R., Zhang, L., Chartier, D., Leung, T. K., and Nattel, S. (2005). Atrial Tachycardia Remodeling of Pulmonary Vein Cardiomyocytes. *Circulation* 111, 728–735. doi:10.1161/01.CIR.0000155240.05251.D0.

Christ, T., Boknik, P., Wöhrl, S., Wettwer, E., Graf, E. M., Bosch, R. F., et al. (2004). L-Type Ca2+ Current Downregulation in Chronic Human Atrial Fibrillation Is Associated With Increased Activity of Protein Phosphatases. *Circulation* 110, 2651–2657. doi:10.1161/01.CIR.0000145659.80212.6A.

Clayton, R. H., Bernus, O., Cherry, E. M., Dierckx, H., Fenton, F. H., Mirabella, L., et al. (2011). Models of cardiac tissue electrophysiology: Progress, challenges and open questions. *Prog. Biophys. Mol. Biol.* 104, 22–48. doi:10.1016/j.pbiomolbio.2010.05.008.

Colman, M. A., Aslanidi, O. V., Kharche, S., Boyett, M. R., Garratt, C., Hancox, J. C., et al. (2013). Pro-arrhythmogenic effects of atrial fibrillation-induced electrical remodelling: insights from the three-dimensional virtual human atria. *J. Physiol.* 591, 4249–4272. doi:10.1113/jphysiol.2013.254987.

Colman, M. A., Ni, H., Liang, B., Schmitt, N., and Zhang, H. (2017). In silico assessment of genetic variation in KCNA5 reveals multiple mechanisms of human atrial arrhythmogenesis. *PLOS Comput. Biol.* 13, e1005587. doi:10.1371/journal.pcbi.1005587.

Courtemanche, M., Ramirez, R. J., and Nattel, S. (1998). Ionic mechanisms underlying human atrial action potential properties: insights from a mathematical model. *Am. J. Physiol. - Heart Circ. Physiol.* 275, H301–H321.

Datino, T., Macle, L., Qi, X.-Y., Maguy, A., Comtois, P., Chartier, D., et al. (2010). Mechanisms by Which Adenosine Restores Conduction in Dormant Canine Pulmonary Veins. *Circulation* 121, 963–972. doi:10.1161/CIRCULATIONAHA.109.893107.

Dobrev, D., Graf, E., Wettwer, E., Himmel, H. M., Hála, O., Doerfel, C., et al. (2001). Molecular Basis of Downregulation of G-Protein–Coupled Inward Rectifying K+ Current (IK,ACh) in Chronic Human Atrial Fibrillation. *Circulation* 104, 2551–2557. doi:10.1161/hc4601.099466.

Dobrev, D., and Ravens, U. (2003). Remodeling of cardiomyocyte ion channels in human atrial fibrillation. *Basic Res. Cardiol.* 98, 137–148. doi:10.1007/s00395-003-0409-8.

Ehrlich, J. R., Cha, T.-J., Zhang, L., Chartier, D., Melnyk, P., Hohnloser, S. H., et al. (2003). Cellular electrophysiology of canine pulmonary vein cardiomyocytes: action potential and ionic current properties. *J. Physiol.* 551, 801–813. doi:10.1113/jphysiol.2003.046417.

El Harchi, A., McPate, M. J., Zhang, Y. H., Zhang, H., and Hancox, J. C. (2010). Action potential clamp and mefloquine sensitivity of recombinant "I KS" channels incorporating the V307L KCNQ1 mutation. *J. Physiol. Pharmacol. Off. J. Pol. Physiol. Soc.* 61, 123–131.

Fabbri, A., Fantini, M., Wilders, R., and Severi, S. (2017). Computational analysis of the human sinus node action potential: model development and effects of mutations. *J. Physiol.* 595, 2365–2396. doi:10.1113/JP273259.

Fedorov, V. V., Glukhov, A. V., Chang, R., Kostecki, G., Aferol, H., Hucker, W. J., et al. (2010). Optical Mapping of the Isolated Coronary-Perfused Human Sinus Node. *J. Am. Coll. Cardiol.* 56, 1386–1394. doi:10.1016/j.jacc.2010.03.098.

Feng, J., Yue, L., Wang, Z., and Nattel, S. (1998). Ionic Mechanisms of Regional Action Potential Heterogeneity in the Canine Right Atrium. *Circ. Res.* 83, 541–551. doi:10.1161/01.RES.83.5.541.

Gong, D., Zhang, Y., Cai, B., Meng, Q., Jiang, S., Li, X., et al. (2008). Characterization and comparison of Na+, K+ and Ca2+ currents between myocytes from human atrial right appendage and atrial septum. *Cell. Physiol. Biochem. Int. J. Exp. Cell. Physiol. Biochem. Pharmacol.* 21, 385–394. doi:10.1159/000129631.

Grandi, E., Pandit, S. V., Voigt, N., Workman, A. J., Dobrev, D., Jalife, J., et al. (2011). Human Atrial Action Potential and Ca2+ ModelNovelty and Significance: Sinus Rhythm and Chronic Atrial Fibrillation. *Circ. Res.* 109, 1055–1066. doi:10.1161/CIRCRESAHA.111.253955.

Hong, K., Piper, D. R., Diaz-Valdecantos, A., Brugada, J., Oliva, A., Burashnikov, E., et al. (2005). De novo KCNQ1 mutation responsible for atrial fibrillation and short QT syndrome in utero. *Cardiovasc. Res.* 68, 433–440. doi:10.1016/j.cardiores.2005.06.023.

Kang, J., Chen, X.-L., Wang, L., and Rampe, D. (2001). Interactions of the Antimalarial Drug Mefloquine with the Human Cardiac Potassium Channels KvLQT1/minK and HERG. *J. Pharmacol. Exp. Ther.* 299, 290–296.

Katoh, H., Shinozaki, T., Baba, S., Satoh, S., Kagaya, Y., Watanabe, J., et al. (2005). Monophasic Action Potential Duration at the Crista Terminalis in Patients With Sinus Node Disease. *Circ. J.* 69, 1361–1367. doi:10.1253/circj.69.1361.

Kharche, S., Adeniran, I., Stott, J., Law, P., Boyett, M. R., Hancox, J. C., et al. (2012). Pro-arrhythmogenic effects of the S140G KCNQ1 mutation in human atrial fibrillation – insights from modelling. *J. Physiol.* 590, 4501–4514. doi:10.1113/jphysiol.2012.229146.

Kim, B.-S., Kim, Y.-H., Hwang, G.-S., Pak, H.-N., Lee, S. C., Shim, W. J., et al. (2002). Action potential duration restitution kinetics in human atrial fibrillation. *J. Am. Coll. Cardiol.* 39, 1329–1336. doi:10.1016/S0735-1097(02)01760-6.

Koumi, S., Sato, R., Katori, R., Hisatome, I., Nagasawa, K., and Hayakawa, H. (1992). Sodium channel states control binding and unbinding behaviour of antiarrhythmic drugs in cardiac myocytes from the guinea pig. *Cardiovasc. Res.* 26, 1199–1205. doi:10.1093/cvr/26.12.1199.

Krueger, M. W., Schmidt, V., Tobón, C., Weber, F. M., Lorenz, C., Keller, D. U. J., et al. (2011). Modeling Atrial Fiber Orientation in Patient-Specific Geometries: A Semi-automatic Rule-Based Approach. in *Functional Imaging and Modeling of the Heart* Lecture Notes in Computer Science. (Springer, Berlin, Heidelberg), 223–232. doi:10.1007/978-3-642-21028-0_28.

Krueger, M. W., Seemann, G., Rhode, K., Keller, D. U. J., Schilling, C., Arujuna, A., et al. (2013). Personalization of Atrial Anatomy and Electrophysiology as a Basis for Clinical Modeling of Radio-Frequency Ablation of Atrial Fibrillation. *IEEE Trans. Med. Imaging* 32, 73–84. doi:10.1109/TMI.2012.2201948.

Lemery, R., Birnie, D., Tang, A. S. l., Green, M., Gollob, M., Hendry, M., et al. (2007). Normal Atrial Activation and Voltage During Sinus Rhythm in the Human Heart: An Endocardial and Epicardial Mapping Study in Patients with a History of Atrial Fibrillation. *J. Cardiovasc. Electrophysiol.* 18, 402–408. doi:10.1111/j.1540-8167.2007.00762.x.

Li, D., Zhang, L., Kneller, J., and Nattel, S. (2001). Potential Ionic Mechanism for Repolarization Differences Between Canine Right and Left Atrium. *Circ. Res.* 88, 1168–1175. doi:10.1161/hh1101.091266.

McPate, M. J., Duncan, R. S., Hancox, J. C., and Witchel, H. J. (2008). Pharmacology of the short QT syndrome N588K-hERG K+ channel mutation: differential impact on selected class I and class III antiarrhythmic drugs. *Br. J. Pharmacol.* 155, 957–966. doi:10.1038/bjp.2008.325.

Moreno, J. D., Lewis, T. J., and Clancy, C. E. (2016). Parameterization for In-Silico Modeling of Ion Channel Interactions with Drugs. *PLOS ONE* 11, e0150761. doi:10.1371/journal.pone.0150761.

Neef, S., Dybkova, N., Sossalla, S., Ort, K. R., Fluschnik, N., Neumann, K., et al. (2010). CaMKII-Dependent Diastolic SR Ca2+ Leak and Elevated Diastolic Ca2+ Levels in Right Atrial Myocardium of Patients With Atrial Fibrillation. *Circ. Res.* 106, 1134–1144. doi:10.1161/CIRCRESAHA.109.203836.

Nenov, N. I., Crumb, W. J., Pigott, J. D., Harrison, L. H., and Clarkson, C. W. (1998). Quinidine Interactions With Human Atrial Potassium Channels. *Circ. Res.* 83, 1224–1231. doi:10.1161/01.RES.83.12.1224.

Ng, J., Kadish, A. H., and Goldberger, J. J. (2006). Effect of electrogram characteristics on the relationship of dominant frequency to atrial activation rate in atrial fibrillation. *Heart Rhythm* 3, 1295–1305. doi:10.1016/j.hrthm.2006.07.027.

Ni, H., Whittaker, D., Wang, W., Giles, W., Narayan, S., and Zhang, H. (2017). Synergistic anti-arrhythmic effects in human atria with combined use of sodium blockers and acacetin. *Front. Physiol.* 8. doi:10.3389/fphys.2017.00946.

Pau, D., Workman, A. J., Kane, K. A., and Rankin, A. C. (2007). Electrophysiological and arrhythmogenic effects of 5-hydroxytryptamine on human atrial cells are reduced in atrial fibrillation. *J. Mol. Cell. Cardiol.* 42, 54–62. doi:10.1016/j.yjmcc.2006.08.007.

Paul, A. A., Witchel, H. J., and Hancox, J. C. (2002). Inhibition of the current of heterologously expressed HERG potassium channels by flecainide and comparison with quinidine, propafenone and lignocaine. *Br. J. Pharmacol.* 136, 717–729. doi:10.1038/sj.bjp.0704784.

Plonsey, R., and Barr, R. C. (2013). *Bioelectricity: A Quantitative Approach*. Springer Science & Business Media.

Qu, Z., Weiss, J. N., and Garfinkel, A. (1999). Cardiac electrical restitution properties and stability of reentrant spiral waves: a simulation study. *Am. J. Physiol. - Heart Circ. Physiol.* 276, H269–H283.

Restier, L., Cheng, L., and Sanguinetti, M. C. (2008). Mechanisms by which atrial fibrillation-associated mutations in the S1 domain of KCNQ1 slow deactivation of IKs channels. *J. Physiol.* 586, 4179–4191. doi:10.1113/jphysiol.2008.157511.

Schotten, U., Greiser, M., Benke, D., Buerkel, K., Ehrenteidt, B., Stellbrink, C., et al. (2002). Atrial fibrillation-induced atrial contractile dysfunction: a tachycardiomyopathy of a different sort. *Cardiovasc. Res.* 53, 192–201. doi:10.1016/S0008-6363(01)00453-9.

Seebohm, G., Lerche, C., Busch, A., and Bachmann, A. (2001). Dependence of IKs biophysical properties on the expression system. *Pflüg. Arch.* 442, 891–895. doi:10.1007/s004240100608.

Seemann, G., Höper, C., Sachse, F. B., Dössel, O., Holden, A. V., and Zhang, H. (2006). Heterogeneous three-dimensional anatomical and electrophysiological model of human atria. *Philos. Trans. R. Soc. Lond. Math. Phys. Eng. Sci.* 364, 1465–1481. doi:10.1098/rsta.2006.1781.

Shaker potassium channel gating. II: Transitions in the activation pathway (1994). *J. Gen. Physiol.* 103, 279–319.

Shaker potassium channel gating. III: Evaluation of kinetic models for activation (1994). *J. Gen. Physiol.* 103, 321–362.

Silva, J., and Rudy, Y. (2005). Subunit Interaction Determines IKs Participation in Cardiac Repolarization and Repolarization Reserve. *Circulation* 112, 1384–1391. doi:10.1161/CIRCULATIONAHA.105.543306.

Van Wagoner, D. R., and Nerbonne, J. M. (2000). Molecular Basis of Electrical Remodeling in Atrial Fibrillation. *J. Mol. Cell. Cardiol.* 32, 1101–1117. doi:10.1006/jmcc.2000.1147.

Verkerk, A. O., Wilders, R., Borren, M. M. G. J. van, Peters, R. J. G., Broekhuis, E., Lam, K., et al. (2007). Pacemaker current (If) in the human sinoatrial node. *Eur. Heart J.* 28, 2472–2478. doi:10.1093/eurheartj/ehm339.

Voigt, N., Li, N., Wang, Q., Wang, W., Trafford, A. W., Abu-Taha, I., et al. (2012). Enhanced Sarcoplasmic Reticulum Ca2+ Leak and Increased Na+-Ca2+ Exchanger Function Underlie Delayed Afterdepolarizations in Patients With Chronic Atrial FibrillationClinical Perspective. *Circulation* 125, 2059–2070. doi:10.1161/CIRCULATIONAHA.111.067306.

Wagoner, D. R. V., Pond, A. L., Lamorgese, M., Rossie, S. S., McCarthy, P. M., and Nerbonne, J. M. (1999). Atrial L-Type Ca2+ Currents and Human Atrial Fibrillation. *Circ. Res.* 85, 428–436. doi:10.1161/01.RES.85.5.428.

Wagoner, D. R. V., Pond, A. L., McCarthy, P. M., Trimmer, J. S., and Nerbonne, J. M. (1997). Outward K+ Current Densities and Kv1.5 Expression Are Reduced in Chronic Human Atrial Fibrillation. *Circ. Res.* 80, 772–781. doi:10.1161/01.RES.80.6.772.

Wang, Z., Fermini, B., and Nattel, S. (1994). Rapid and slow components of delayed rectifier current in human atrial myocytes. *Cardiovasc. Res.* 28, 1540–1546.

Whittaker, D. G., Ni, H., Benson, A. P., Hancox, J. C., and Zhang, H. (2017a). Computational Analysis of the Mode of Action of Disopyramide and Quinidine on hERG-Linked Short QT Syndrome in Human Ventricles. *Front. Physiol.* 8. doi:10.3389/fphys.2017.00759.

Whittaker, D. G., Ni, H., Harchi, A. E., Hancox, J. C., and Zhang, H. (2017b). Atrial arrhythmogenicity of KCNJ2 mutations in short QT syndrome: Insights from virtual human atria. *PLOS Comput. Biol.* 13, e1005593. doi:10.1371/journal.pcbi.1005593.

Workman, A. J., Kane, K. A., and Rankin, A. C. (2001). The contribution of ionic currents to changes in refractoriness of human atrial myocytes associated with chronic atrial fibrillation. *Cardiovasc. Res.* 52, 226–235. doi:10.1016/S0008-6363(01)00380-7.

Wu, L., Guo, D., Li, H., Hackett, J., Yan, G.-X., Jiao, Z., et al. (2008). Role of late sodium current in modulating the proarrhythmic and antiarrhythmic effects of quinidine. *Heart Rhythm* 5, 1726–1734. doi:10.1016/j.hrthm.2008.09.008.

Zhang, Y., and Hancox, J. (2002). Mode-dependent inhibition by quinidine of Na+–Ca2+ exchanger current from guinea-pig isolated ventricular myocytes. *Clin. Exp. Pharmacol. Physiol.* 29, 777–781. doi:10.1046/j.1440-1681.2002.03731.x.
